# The Language of Inequality: Evidence Economic Inequality Increases Wealth Category Salience

**DOI:** 10.1177/01461672211036627

**Published:** 2021-08-05

**Authors:** Kim Peters, Jolanda Jetten, Porntida Tanjitpiyanond, Zhechen Wang, Frank Mols, Maykel Verkuyten

**Affiliations:** 1 University of Exeter, UK; 2 The University of Queensland, Brisbane, Australia; 3 Utrecht University, The Netherlands

**Keywords:** economic inequality, self-categorization, language, wealth, rich, poor

## Abstract

There is evidence that in more economically unequal societies, social relations are more strained. We argue that this may reflect the tendency for wealth to become a more fitting lens for seeing the world, so that in economically more unequal circumstances, people more readily divide the world into “the haves” and “have nots.” Our argument is supported by archival and experimental evidence. Two archival analyses reveal that at times of greater inequality, books in the United Kingdom and the United States and news media in English-speaking countries were more likely to mention the rich and poor. Three experiments, two preregistered, provided evidence for the causal role of economic inequality in people’s use of wealth categories when describing life in a fictional society; effects were weaker when examining real economic contexts. Thus, one way in which inequality changes the world may be by changing how we see it.

Everybody’s talkin’ about hard times . . .

Fat cats on Wall Street

They got a bailout

While somebody else got to wait.

Prince (2009)

There is evidence that countries that have higher levels of economic inequality have greater levels of social division (for a review see, d’Hombres et al., 2012). We suggest that the observations of the artist Prince, above, may shed light on why this could be. In particular, his lyrics suggest that the prevailing economic conditions of 2009 got Americans talking about the “haves” (who got bailouts) and the “have nots” (who did not get any assistance). While an essential part of those prevailing conditions was the global financial crisis, what may be even more important is what had come before it: 35 years of relentless growth in the gap between the incomes of those at the top and bottom of American society. As we will argue in greater detail below, there is reason to believe that this (ever increasing) economic inequality may have made wealth a more fitting lens for understanding the world. To the extent that it did, people could be expected to more readily divide—and describe—the world in terms of the haves and have nots; to the extent that this unequal allocation is perceived as unfair and illegitimate, these processes could ultimately foment social division and intergroup conflict.

There is initial evidence that growing inequality is associated with a range of societal consequences, including reductions in trust and social capital, and increases in violence and social unrest (Fajnzylber et al., 2002; Uslaner & Brown, 2005). While this is suggestive of the *possibility* that inequality may have social psychological consequences, this has received relatively little attention to date (for a discussion, see Jetten & Peters, 2019). In this article, we aim to contribute to the social psychological analysis of economic inequality by examining a basic proposition, articulated by Jetten et al. (2017), that inequality increases people’s tendencies to see the world through a lens of wealth. This proposition is an important one because, as work in the social identity tradition has demonstrated (Tajfel & Turner, 1979; Turner & Oakes, 1986), the tendency for people to categorize self and others into social groups is a building block for a broad range of group-related social behaviors and societal outcomes (positive and negative). In other words, if economic inequality is found to affect people’s perceptions of the shape of their social world, then it can be expected to shape the way in which they act in it.

The proposition that economic inequality will make wealth a more fitting basis for categorizing the social world has been alluded to in existing work. For instance, Wilkinson and Pickett (2009) argued that if

inequalities are bigger, . . . where each one of us is placed becomes more important. Greater inequality is likely to be accompanied by increased status competition and increased status anxiety. It is not simply that where the stakes are higher each of us worries more about where he or she comes. It is also that we are likely to pay more attention to social status in how we assess each other. (p. 44)

Along the same lines, in explaining the reduction in Americans’ saving levels toward the end of the last century, Schor (1998) suggested that rising economic inequality has increased the salience of very wealthy members of society which has in turn fuelled “competitive consumption” by the middle class.

Importantly, there are theoretical reasons for expecting that greater economic inequality will increase the salience of wealth categories (for related ideas, see Callero, 2014). In particular, self-categorization theory (Turner et al., 1987) proposes that people’s tendencies to see their social world in terms of social categories will depend on the extent to which those categories provide a *fitting* basis for social categorization (Bruner, 1957; Oakes et al., 1994). Of particular importance is the notion of comparative fit, which argues that a category is more likely to be used to parse the social world when people perceive that the differences between those who belong to the category and those who do not are greater than the differences among those who belong to the category (Turner, 1987, 1999). There is a large body of empirical work that shows that comparative fit can affect the salience of social categories (e.g., Haslam & Turner, 1992; Oakes et al., 1991). Furthermore, Pietraszewski et al. (2015) found that when the experimental context increased the comparative fit of political group membership, participants were more likely to treat people who belonged to the same political party as more similar (and interchangeable) than people who belonged to the same race.

Although the possibility that economic inequality may increase the salience of wealth categories has not, to our knowledge, been directly tested, there is some evidence that economic inequality affects perceptions of others. Primary among this is a growing body of work that suggests that economic inequality affects people’s stereotypes about a range of groups in society, including the rich and poor. For instance, in a broader cross-national study of the content of a range of societal stereotypes, Durante et al. (2013) found that in more economically unequal countries, the rich were viewed as especially cold, while the poor were viewed as especially incompetent. Along the same lines, Heiserman and Simpson (2017) found that participants who were asked to join a more economically unequal de novo society rated the competence deficit of the poor (relative to the rich) as greater than participants who were asked to join a more economically equal society.

This work suggests that as economic inequality increases, income and wealth are more likely to become a fitting basis for categorizing self and others in society—in this case, increasing the extremity of the stereotypes of the rich and poor. And to the extent that people see the world through a lens of wealth, they can be expected to describe their world along the same lines. If so, this points to one way in which people’s beliefs about the importance of wealth-based divisions in society can become widely shared. The basis for this expectation is provided by work into cultural dynamics (e.g., Kashima et al., 2007) which argues that the language that people use, and ideas that people share, is more likely to become incorporated into a society’s body of shared knowledge. This idea is supported by work that has shown that the content of people’s communications is reflected in a range of cultural level beliefs, including stereotypes (e.g., Kashima & Kashima, 2003; Schaller et al., 2002).

In this article, we aim to provide the first formal test of Jetten et al.’s (2017) hypothesis that the salience of wealth-based social categories will increase with economic inequality. To the extent that wealth categories become more salient, we expect that people will be more likely to spontaneously use these categories when describing their social world and will place greater importance on information about a person’s wealth. This provides the basis for our hypotheses:

**Hypothesis 1 (H1):** When inequality is greater, people will make greater reference to wealth categories in language.
**Hypothesis 2 (H2):** When inequality is greater, people will rate others’ wealth-related attributes as more important.

## Study Overview

We report the results of five studies that tested these hypotheses. Studies 1 and 2 used archival methods to examine whether there is an association between a country’s level of economic inequality over time and the prevalence of wealth category words (like “rich” and “poor”) in cultural artifacts like books and news media. Studies 3a and 3b use an experimental design to test the causal impact of being placed in a more or less unequal social context on people’s spontaneous use of wealth category words and concern about the wealth of others. Finally, Study 4 aimed to move beyond a de novo context to see if manipulating people’s perceptions of inequality in their own society has similar effects. For Studies 3b and 4, we preregistered the study design, a preplanned stopping rule, inclusion/exclusion criteria, hypotheses, and analysis plan (Study 3b: https://osf.io/pz3af/; Study 4: https://osf.io/2wxja/). The Supplemental Documents include all materials, data, and syntax as well as a summary of Supplemental Analyses (SA) to those presented in text and the results of a survey (Study 5) of the association between perceptions of economic inequality and wealth category salience. Together, these studies provide correlational and causal evidence in favor of H1, and more limited evidence for H2. Findings are stronger in the lab than in real economic contexts. The data that support the findings of this study are openly available at https://osf.io/m2htd/.

## Study 1

Study 1 provides our first archival test of the association between economic inequality and wealth category references. We specifically examine the association between these words in English language books published in the United States and the United Kingdom over a period of almost 100 years.

### Method

#### Measures

To estimate the prevalence of wealth category words in books that were published in English in the United States and the United Kingdom, we used the Google Books Ngram Viewer (https://books.google.com/ngrams/info#). This tool is able to estimate the prevalence of words of interest (as a percentage of all words) among the approximately 40 million titles that have been digitized by Google. We conducted our search for wealth category words from 1910 to 2008 in the United Kingdom and United States. This temporal range was determined by limits on the availability of data on historical economic inequality (available from 1910 to 2013) and from Google Books (the archive runs up to 2008).

The wealth category word dictionary consisted of 35 adjectives relating to a person’s high or low wealth. To compile the dictionary, we conducted online thesaurus searches to identify synonyms of the words “rich” and “poor.” From an initial list of 74 adjectives (see SA), we removed words that were very rare and that had multiple nonwealth-related meanings (e.g., fat, swimming, meager, insufficient). The final dictionary included 17 high-wealth words (rich, upper class, millionaire, wealth, loaded, privileged, affluent, prosperous, posh, flush, new money, well off, well heeled, well to do, high class, old money, nouveau riche) and 18 low-wealth words (poor, impoverished, destitute, indigent, needy, penniless, poverty stricken, underprivileged, insolvent, beggared, deprived, disadvantaged, disenfranchised, lower class, broke, impecunious, penurious, cash strapped).

We collected estimates of two economic indicators: economic inequality and, as a control, per capita gross domestic product (GDP). As a measure of inequality, we used Piketty’s (2014) calculations of the share of income that was owned by the wealthiest decile of the population in the United Kingdom and United States between 1910 and 2008 (data are missing for 3 years in the United Kingdom). As a measure of economic growth in these countries over the same period, we used Roser’s (2013) estimates of per capita GDP. We also collected data on each country’s poverty rate from the U.S. Census Bureau (available from 1959) and U.K. Fiscal Studies (available from 1961) and used this as an additional control in our robustness analyses. To contend with the fact that our variables have greatly different scales (from very small percentages to very large dollar values), we standardized our variables into *z* scores.

### Results

**Figure 1.**
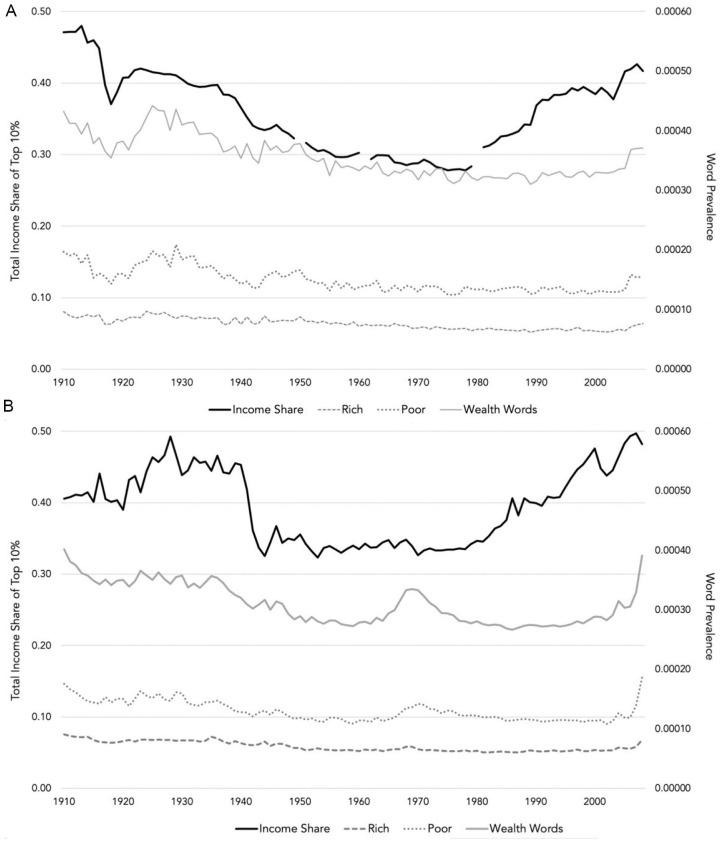
Graphs depicting inequality and wealth category word prevalence in English language books published in the United Kingdom and United States between 1910 and 2008, Study 1. (A) United Kingdom and (B) United States. *Note.* Inequality measured as the share of the income earned by the top decile of the population. Word prevalence measured as the percentage of all words in English language books published in the United States or United Kingdom indexed by Google books that reference wealth categories, or the words “rich” or “poor.”

Figure 1 graphs the levels of economic inequality in the United States and United Kingdom, the average prevalence of all wealth category words as well as the prototypical words “rich” and “poor” between 1910 and 2008. This figure reveals that the percentage of income owned by the top decile in these countries was relatively high in the first part of the 20th century, before declining after the Second World War, and then picking up again from the 1980s onwards. The prevalence of wealth category words appears to show a similar pattern in that these words were more prevalent in the first part of the 20th century before becoming, relatively, less common. However, the usage of these words does not start to increase again until the start of the 21st century.

To test our claim that there would be more references to wealth in language when economic inequality is high, we regressed the prevalence of the 35 wealth category words onto the income share owned by the top decile for the United Kingdom and the United States in turn (higher values point to greater inequality). We also included covariates to control for the possibility that the use of wealth category terms may be subject to temporal changes (in years, zeroed at 1910) and affected by a country’s wealth (GDP, standardized). To account for the lack of independence that may result from the nested structure of the data (years within words), we ran two-level random effects models.

Table 1 contains the unstandardized regression coefficients. In Step 1, we regressed the percentage of wealth category references onto year and income share. This revealed a significant positive association between income share and the prevalence of wealth category referencing words in both countries, which is consistent with H1 and our expectation that greater inequality will be accompanied by greater use of wealth categories in language. There were also significant negative effects of year in both countries, indicating that, controlling for inequality, wealth category words became less common over time. To examine the equivalent association between GDP and word prevalence, in Step 2, we replaced the income share term with that for GDP. This revealed significant positive effects of GDP, which suggests that when these countries were wealthier, it was more likely that their books would refer to wealth categories. Finally, to see whether inequality accounted for unique variance in word prevalence after controlling for GDP, in Step 3, we added the income share term. This led to significant improvement in model fit for the United States, χ^2^(1) = 11.74, *p* < .001, and the United Kingdom, χ^2^(1) = 9.28, *p* = .002. Importantly, in line with H1, inequality was again a significant positive predictor in both countries. GDP remained a positive predictor in the United States, but not the United Kingdom.

**Table 1.** Unstandardized Coefficients of the Association Between Changes in Economic Indicators and Changes in Percentage of Wealth Category Words in Books, Study 1.

	United States			United Kingdom		
Predictor	Step 1	Step 2	Step 3	Step 1	Step 2	Step 3
Year	−.001*** (.000)	−.002*** (.000)	−.002*** (.000)	−.001*** (.000)	−.002*** (.000)	−.001 (.001)
GDP		.048*** (.008)	.025* (.010)		.043*** (.009)	−.006 (.018)
Inequality	.017*** (.003)		.011** (.003)	.017*** (.003)		.018** (.005)
Constant	.024 (.156)	.104 (.157)	.065 (.157)	.075 (.177)	.145 (.178)	.065 (.180)
Observations	3,465	3,465	3,465	3,360	3,360	3,360

*Note.* Economic variables are standardized to *z* values; year is zeroed at 1910; standard errors are presented in brackets. GDP = gross domestic product.

* *p* < .050. **p < .010. ***p < .001.

**Table 2.** Factiva and Gini Coverage by Countries and Regions, Study 2.

Country	Factiva coverage		Gini coefficient coverage		
	Start year	Valid years	Valid w. Gini	Gini type	Gini source
Australia	1986	31	71%	Disposable	Australian Bureau of Statistics
Canada	1977	39	97%	Gross Disposable	Statistics Canada
Hong Kong	1984	27	22%	Gross^a,b^	Census and Statistics Department, Hong Kong
India	1986	22	14%	Gross^c^	World Income Inequality
Ireland	1981	23	96%	Disposable	Eurostat 2018, European Commission 2005
Malaysia	1985	24	42%	Gross^a^	Department of Statistics Malaysia
New Zealand	1986	28	71%	Disposable^d^	Ministry of Social Development, New Zealand
The Philippines	1995	21	33%	Gross^a^	Philippine Statistics Authority
Singapore	1984	28	61%	Gross Disposable	Singapore Department of Statistics
South Africa	1992	18	33%	Gross^a^	Statistics South Africa
United Kingdom	1981	36	100%	Gross Disposable	U.K. Office for National Statistics
United States	1975	38	100%	Gross	U.S. Census Bureau

*Note.* Start year = first year of Factiva coverage; valid years = number of years with more than 10,000 articles; valid w. Gini = percentage of valid years with a Gini coefficient.

a Not equivalence adjusted. ^b^Original household income. ^c^Expenditure, not equivalence. ^d^Before housing costs deducted.

Analyses reported in SA show that these results are robust to the inclusion of poverty level as an additional control for the United Kingdom, but not the United States.

### Discussion

This study provides evidence that is consistent with H1. In years when the richest 10% of the U.K. and U.S. populations owned more income than they had before, books that were published in these countries included more references to the rich and poor than they had before. We also observed a positive association between a country’s wealth (as measured by GDP) and the prevalence of wealth category adjectives in the United States, suggesting that broader economic circumstances may also factor into wealth category salience.

While this study provides a promising hint that economic inequality may increase the salience of wealth categories, it is limited by our focus on only two countries, the United Kingdom and the United States, as well as our focus on one particular kind of cultural artifact: books. Study 2 addresses these limitations by using archival methods that focus on media publications across a greater number of countries. Specifically, in this study, we examine whether levels of economic inequality in 12 different countries and regions are associated with the prevalence of wealth category words in their newspapers and magazines.

## Study 2

### Method

We used Factiva, a Dow Jones database that archives content from more than 23,000 media sources, to obtain an estimate of the prevalence of wealth category words in countries that have high levels of spoken English. Our sample included 12 countries and regions with a minimum of 100,000 English language articles archived in the decade from 2006 (these estimates were obtained by searching for all articles that included the word “the”). Of these countries, six were anglosphere countries (the United Kingdom, the United States, Australia, New Zealand, Canada, and Ireland), and the remainder had English as an official language (Singapore, India, the Philippines, Malaysia, Hong Kong, and South Africa).

We used visual-basic script (VBS)-based programming software (a Microsoft scripting language that runs on webpages) to obtain an annual count for each country of the English language articles that contained each of the words in our wealth category dictionary. Because the number of articles published each year in each country varies greatly, we calculated the percentage of articles that contain a given wealth category word (by dividing it by the number of articles containing the word “the”). Therefore, our dependent variable in the analysis that follows is the percentage of articles published in a given country in a given year that reference a wealth category word.

As in Study 1, we included two economic indicators as predictors: Gini coefficient and GDP. As can be seen in Table 2, there were variations in the ways in which Gini coefficients were calculated across countries and also in the number of years for which we were able to obtain coefficients. We obtained each country’s annual GDP in U.S. dollars from the World Bank database. To check the robustness of our findings, we also collected additional GDP indices, including GDP growth and GDP per capita, and national poverty data.

### Results

**Figure 2.**
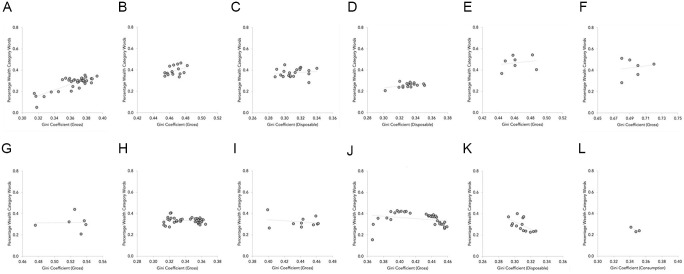
Scatter graphs depicting the association of Gini coefficient and percentage of wealth referencing articles in each country, Study 2. (A) The United Kingdom, (B) Singapore, (C) Ireland, (D) New Zealand, (E) the Philippines (F) South Africa, (G) Hong Kong, (H) Canada, (I) Malaysia, (J) the United States, (K) Australia, and (L) India.

The association between each country’s Gini coefficient and the percentage of media articles using wealth category words is represented in Figure 2. From this, it can be seen that the strength of the association varies somewhat across countries, but is predominantly positive (with Australia and the United States as notable exceptions): the higher the Gini coefficient, the more the wealth-related words are used.

To test our expectation that the prevalence of wealth category words within a country’s media would increase with increases in that country’s level of economic inequality, we regressed the percentage of wealth category words onto the Gini coefficient (standardized). Where we were able to obtain two Gini estimates for a country, we used the gross Gini coefficient. We also included covariates to control for the possibility that the use of wealth category terms may be subject to temporal changes (in years, zeroed at 1975) and affected by a country’s wealth (GDP, standardized). To minimize the possibility that the results would be biased by years that had a low base rate of articles, we restricted our analysis to years for which a country had 10,000 or more articles. To account for the lack of independence that may result from the nested structure of the data (years within words within countries), we ran three-level random effects models. To control for the possibility of unobserved higher level endogeneity, we incorporated clustered random error terms for the country-level predictors (i.e., Gini and GDP; Antonakis et al., 2021; Mundlak, 1978).

Table 3 contains the regression unstandardized coefficients. In Step 1, we regressed the percentage of wealth category references onto year, the Gini coefficient, and its clustered random error term. This revealed a significant positive association between the Gini coefficient and the prevalence of wealth category referencing articles, which is consistent with H1 and our expectation that greater inequality will be accompanied by greater use of wealth categories in language. To examine the equivalent association between GDP and word prevalence, in Step 2, we replaced the Gini terms with those for GDP. Unlike Study 1, this revealed a significant negative effect of GDP, which suggests that when countries are wealthier, their media are less likely to refer to wealth categories; there was also a positive significant effect of year, such that wealth category references became more prevalent over time. Finally, to see whether inequality accounted for unique variance in word prevalence after controlling for GDP, in Step 3, we added the Gini coefficient terms. This led to a significant improvement in model fit, χ^2^(2) = 37.30, *p* < .001, and in line with H1, the Gini coefficient was again a significant positive predictor.

**Table 3.** Unstandardized Coefficients of the Association Between Economic Indicators and Percentage of Articles With Wealth Category References, Study 2.

Predictor	Step 1	Step 2	Step 3
Year	−.000 (.000)	.001*** (.000)	.001** (.000)
GDP		−.027*** (.004)	−.038*** (.004)
GDP (CRE)		.030 (.060)	.042 (.050)
Gini	.025* (.010)		.067*** (.011)
Gini (CRE)	.005 (.029)		−.038 (.030)
Constant	0.336*** (.039)	0.300*** (.039)	0.300*** (.039)
Observations	7,420	7,420	7,420

*Note.* GDP = gross domestic product; CRE = clustered random error term.

* *p* < .050. **p < .010. ***p < .001.

Robustness analyses, reported in SA, show that results broadly replicate for different measures of Gini and GDP and also when poverty is included as a control.

### Discussion

We find additional evidence of a positive association between the use of wealth category words and the level of inequality within a country such that when levels of inequality (as measured by the Gini coefficient) are higher so too is the frequency with which that country’s media references wealth categories. Unlike Study 1, we observed a negative association between a country’s wealth (as measured by GDP) and the prevalence of wealth category adjectives (it is important to note, though, that in the analyses reported in SA, the direction of this effect varied as a function of the specific countries that were examined). Across both studies, therefore, we observe little consistency in the association between national wealth and wealth references.

It is interesting to observe that across both studies, the United Kingdom provides the most consistent evidence that inequality and wealth category prevalence are positively associated, while the United States provides more mixed evidence for this association. This suggests that there may be important unobserved country-level factors (for instance, associated with cultural beliefs or ideology) that may condition the extent to which societal inequality comes to be reflected in people’s descriptions of their social world.

While the Study 1 and 2 findings provide consistent and highly suggestive evidence that the distribution of income within a country increases the salience of wealth categories among its citizens, these studies are not able to speak to the *causal* impact of inequality on wealth category salience. This raises the possibility that an unmeasured third variable could account for the association between these variables, such as an increasingly capitalist economy, which could simultaneously produce inequality and increase the salience of wealth categories. To address this limitation, Studies 3a and 3b manipulate perceptions of inequality and observe the impact of this on the salience of wealth categories. Specifically, in these studies, we ask participants to imagine joining a fictional society that is either more or less economically unequal. We then assess their tendencies to spontaneously refer to wealth categories when describing what their own life and the life of another individual would be like in that society as well as how much importance they place on knowing the wealth of other citizens. The latter measure allows us to test H2. These studies use a U.S. sample; although the archival evidence for the United States was mixed, we had no a priori basis for expecting that cultural differences would condition the impact of our manipulation of inequality in a de novo society.

## Studies 3a and 3b

### Method

#### Participants

The initial sample of Study 3a consisted of 368 Amazon Mechanical Turk workers who clicked on the survey link and were allocated to a condition. Participants were paid US$1 for their time. We excluded 116 of these participants because they had missing data on the core measures of interest (78 stopped before the first open text response, and the remainder did not respond to the second). Of those without missing data, we excluded a further 16 because they failed at least one of the attention checks and a final 10 who provided a low-quality response to at least one of the open text questions (i.e., reproducing the question or another source, or providing text that was nonsensical).^1^ The number of exclusions did not differ by condition: low inequality 41.1%, high inequality 36.1%, χ^2^(1) = 0.98, *p* = .323

The final Study 3a sample for analytic purposes consisted of 226 participants, slightly exceeding our aim of a minimum *N* = 100 for each of the two conditions. A sensitivity analysis using G*Power (Faul et al., 2009) revealed that a two-tailed independent samples *t* test with an alpha of .05 would have 80% power to detect a small-to-medium effect of *d* = 0.37 and 95% power to detect a medium effect of *d* = 0.48 in this sample. Participants were 35.38 (*SD* = 11.21) years old on average and were about equally likely to be male as female (men *N* = 115). The majority resided in the United States (*N* = 223; missing *N* = 1), were native English speakers (*N* = 218), had some postsecondary education (*N* = 174), and placed themselves around the mid-point of the 10-rung socioeconomic status (SES) ladder (*M* = 5.09, *SD* = 3.93).

To test the robustness of the Study 3a findings, we then conducted a high-powered preregistered direct replication (available at osf.io/pz3af). The initial sample of Study 3b were 665 Amazon Mechanical Turk workers who passed an initial set of four attention checks. Participants were paid US$2.50 for their time. In line with our preregistered plan, we excluded 93 of these participants because they failed to complete one or both of the free response questions, another 94 who failed at least one of the three additional attention checks, and a final 64 who provided a low-quality response to the free response questions. The number of exclusions did not differ by condition: low inequality 39.5%, high inequality 36.0%, χ^2^(1) = 0.90, *p* = .342.

The final Study 3b sample for analytic purposes consisted of 414 participants. This exceeded our target sample of 400 participants, which we selected as having between 60% to 99% power to detect the H1 effects (Study 3a estimates ranged from *r* = .11 to .23) and 80% to 99% power to detect the H2 effects (Study 3a estimates ranged from *d* = 0.27 to .39). The participants were slightly more likely to be male than female (men *N* = 236; other or prefer not to say *N* = 5) and had an average age of 36.03 years (*SD* = 10.74). The majority were native English speakers (*N* = 403), had some postsecondary education (*N* = 318), and placed themselves at about the mid-point on the 10-point SES ladder (*M* = 5.07, *SD* = 1.73).

#### Inequality manipulation

The procedures for Studies 3a and 3b were identical. Participants were introduced to a de novo society, Bimboola, whose citizens were categorized into one of the three different income groups (for a detailed description of this paradigm, see Sánchez-Rodríguez et al., 2019; Sprong et al., 2019). To manipulate perceptions of inequality, participants who were randomly assigned to the low inequality condition (Study 3a *N* = 109; Study 3b *N* = 202) were told that the members of Income Groups 1, 2, and 3 had an annual income of 50, 40, and 30 Bimboolean Dollars (BD), respectively. Participants assigned to the high inequality condition were instead told that the members of these groups earned 77, 40, and 3 BD, respectively. All participants were told that they belonged to Group 2, and consequently were equally wealthy in both conditions. To reinforce the inequality manipulation, participants were then asked to choose a house, car, and holiday from a set of options that the members of each group could afford. As a Group 2 member, participants were able to choose from the sets available to Group 2 and Group 3, but they could not afford any of the options available to Group 1. While the options available for Group 2 members were identical in the two conditions, the options available for Group 1 and Group 3 members were more luxurious and frugal, respectively, in the high and low one.

#### Measures

Participants first completed a comprehension check: “which income group have you been assigned to?”, and a set of seven manipulation checks: “how wealthy is your group?,” “how poor is your group,” “how wealthy is group 1?,” “how wealthy is group 3?,” “how unequal is Bimboola?”, and “how equal is Bimboola?” (reversed; the latter two items were averaged into a measure of perceived inequality, Study 3a *r* = .78, *p* < .001; Study 3b *r* = .87, *p* < .001). These were accompanied by 9-point Likert-type scales (1 = *not at all wealthy/not at all*, 9 = *very wealthy/very much*).^2^

To assess participants’ tendencies to spontaneously reference wealth categories when describing life in Bimboola (note, we use wealth inclusively to mean income as well as capital), we asked participants to complete two free description tasks. The first asked participants to describe what they thought their own life in Bimboola would be like, including their daily activities, social interactions (i.e., who they would encounter and socialize with), and general thoughts and feelings in day-to-day life. The second introduced participants to a citizen of Bimboola and provided them with 21 statements that described their preferences, daily activities, and life circumstances (see Supplemental Documents). In all cases, this individual was said to be divorced with two children, had close family relationships, an interest in musicals and public speaking, and a preference for certain foods. However, six of these statements were varied such that participants were either told that the person was a member of Group 1, with many of the perks of wealth (e.g., expensive car and holidays), or a member of Group 3, with many of the challenges of poverty (e.g., inability to afford a car or holidays). Participants were randomly allocated to read one of the four versions of the citizen descriptions that varied whether the citizen was rich or poor and, orthogonally, whether they were male or female (i.e., John/Jane). After reading through these descriptions, participants were asked to write a short paragraph describing John/Jane’s life in Bimboola (i.e., his or her daily activities, social interactions, and general thoughts and feelings throughout a typical day in Bimboola).

As a further measure of wealth salience, participants were introduced to yet another Bimboolean, whose gender was again randomly varied, and asked to indicate how important they felt different pieces of information would be for getting to know what this individual was like as a person on 5-point Likert-type scales (1 = *strongly no*, 5 = *strongly yes*). Of key concern, participants were asked to rate the importance of knowing about four demographic variables that are more or less directly related to wealth (“income group,” “salary,” “occupation,” and “education”); they were also asked to rate the importance of knowing about another five that are less so (“hobby,” “political view,” “ethnicity,” “religion,” and “age”). Additional exploratory measures, including the foundations of morality, are discussed in SA.

### Results

#### Content coding

The first author and, in Study 3a, the third author, or in Study 3b, a research assistant who was blind to the hypotheses, independently coded participants’ use of wealth categories in their free descriptions. In particular, participants received codes of 1 (otherwise 0) if their responses mentioned their own income group (Group 2 or synonyms, for example, middle class, average income etc.), the wealthy group (Group 1 or synonyms, for example, rich, upper class etc.), and the poor group (Group 3 or synonyms, for example, poor, lower class etc.). Both coders were blind to experimental condition. The first coding round revealed reasonable to high levels of agreement (own life Kappas: Study 3a 0.69–0.86, Study 3b 0.70–0.86; citizen life Kappas: Study 3a 0.57–0.74, Study 3b 0.59–0.74). The second coding round, which involved discussing and independently recoding disagreements, produced very high levels of agreement (own life Kappas: Study 3a 0.93–0.95, Study 3b 0.90–0.95; citizen life Kappas: Study 3a 0.86–0.92, Study 3b 0.90–0.93). Any remaining disagreements were resolved by the first author. In addition, we used the Linguistic Inquiry and Word Count (LIWC) software package (Pennebaker et al., 2007) to see whether the manipulation of inequality affected the frequency of words within the standard software categories.

#### Manipulation check

Table 4 (Study 3a) and Table 5 (Study 3b) contain the means and intercorrelations of the key measures. To check for the effectiveness of our manipulation, we compared participants’ perceptions of Bimboola’s inequality in the high and low inequality conditions using an independent samples *t* test. This showed that, in line with expectations, participants in the high inequality condition perceived that Bimboola was more unequal than those in the low inequality condition in both Study 3a (*M*_high_ = 8.02, *SD* = 1.35 vs. *M*_low_ = 4.17, *SD* = 1.84), *t*(224) = 18.01, *p* < .001, 95% confidence interval (CI) = [3.42, 4.27], *d* = 2.41, and Study 3b (*M*_high_ = 8.19 *SD* = 1.30 vs. *M*_low_ = 3.98, *SD* = 1.82), *t*(412) = 27.19, *p* < .001, 95% CI [3.91, 4.52], *d* = 2.68. Analysis of perceptions of the wealth of each income group is reported in SA and reveals that these were also successfully manipulated.

**Table 4.** Study 3a Variable Means and Intercorrelations.

Measure	*M* (*SD*)	1	2	3	4	5	6	7	8	9	10
Manipulation checks											
1. Bimboola inequality	6.17 (2.51)										
2. Group 2 wealth	5.24 (0.72)	−.09									
3. Group 1 wealth	7.73 (1.72)	.33**	.16*								
4. Group 3 wealth	2.63 (2.13)	−.42**	.12	−.75**							
Wealth category salience											
5. Own life: Group 2 words	0.45 (0.50)	.25**	−.09	.19**	−.18**						
6. Own life: Group 1 words	0.26 (0.44)	.16*	−.15*	.12	−.14*	.48**					
7. Own life: Group 3 words	0.29 (0.46)	.20**	.01	.17*	−.17**	.40**	.62**				
8. Citizen: Group 2 words	0.13 (0.34)	.23**	−.00	.02	.02	.13*	.16*	.19**			
9. Citizen: Group 1 words	0.32 (0.47)	.12	.03	.08	.01	.18**	.13	.18**	.44**		
10. Citizen: Group 3 words	0.34 (0.48)	.26**	.03	.11	−.06	.20**	.23**	.26**	.39**	.08	
11. Income group importance	2.64 (1.17)	.18**	−.04	.07	−.08	.13	.05	.08	.03	.09	.03

*Note. N* = 226. Group 2 = middle class, Group 1 = rich, Group 3 = poor; manipulation checks measured on 9-point scales, wealth category references measured on dichotomous (0/1) scale, income group information importance measured on 5-point scale.

* *p* < .050. **p < .010.

**Table 5.** Study 3b Variable Means and Intercorrelations.

Measure	*M* (*SD*)	1	2	3	4	5	6	7	8	9	10
Manipulation checks											
1. Bimboola inequality	6.00 (2.56)										
2. Group 2 wealth	5.39 (0.92)	−.14**									
3. Group 1 wealth	7.57 (1.79)	.29**	.16*								
4. Group 3 wealth	2.97 (2.26)	−.42**	.35**	−.70**							
Wealth category salience											
5. Own life: Group 2 words	0.64 (0.48)	.19**	−.22**	.09	−.23**						
6. Own life: Group 1 words	0.35 (0.48)	.11*	−.09	.11*	−.16*	.53**					
7. Own life: Group 3 words	0.39 (0.49)	.21**	−.10*	.07	−.16**	.53**	.66**				
8. Citizen: Group 2 words	0.16 (0.37)	.06	−.03	.03	−.10*	.23**	.12*	.13*			
9. Citizen: Group 1 words	0.39 (0.49)	.10*	−.07	.08	−.12*	.17**	.10*	.09	.30**		
10. Citizen: Group 3 words	0.40 (0.49)	.14**	−.13**	.07	−.17**	.20**	.07	.12*	.36**	−.15	
11. Income group importance	2.67 (1.23)	.15**	.14**	.02	.06	−.01	.09	.11*	−.02	.04	−.01

*Note. N* = 407. Group 2 = middle class, Group 1 = rich, Group 3 = poor; manipulation checks measured on 9-point scales, wealth category references measured on dichotomous (0/1) scale, income group information importance measured on 5-point scale.

* *p* < .05. **p < .010.

#### Wealth references

**Figure 3.**
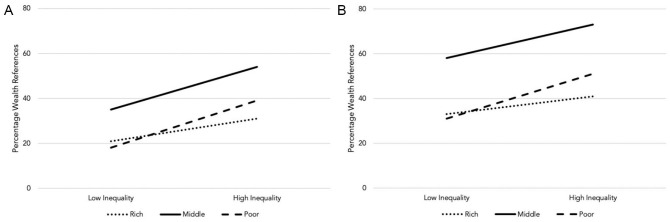
Percentage of participants in Studies 3 and 4 referencing each wealth group when describing their own life when inequality was high and low. Panel (A) graphs the associations for Study 3a and panel (B) graphs the associations for Study 3b.

To test H1, we first used logistic regression to compare the proportion of participants in each condition (dummy variable: high inequality = 1, otherwise 0) who referred to wealth categories in their free descriptions of their own life (see Figure 3). As expected, relative to participants in the low inequality condition, participants in the high inequality condition were more likely to mention Group 2, their own group, in both Study 3a (low 35% vs. high 54%, *B* = 0.78, odds ratio = 2.18), χ^2^(1) = 8.23, *p* = .004, and Study 3b (low 58% vs. high 73%, *B* = 0.64, odds ratio = 1.89), χ^2^(1) = 9.29, *p* = .002. Participants in the high inequality conditions were also more likely to mention Group 3, the poor group, in both Study 3a (low 18% vs. high 39%, *B* = 1.06, odds ratio = 2.88), χ^2^(1) = 11.55, *p* = .001, and Study 3b (low 31% vs. high 51%, *B* = 0.83, odds ratio = 2.29), χ^2^(1) = 16.65, *p* < .001. Descriptively, the same pattern was observed for Group 1, the rich group, but this was not significant in Study 3a (low 21% vs. high 31%, *B* = 0.51, odds ratio = 1.66), χ^2^(1) = 2.71, *p* = .100, and was only marginal in Study 3b, (low 33% vs. high 41%, *B* = 0.34, odds ratio = 1.40), χ^2^(1) = 2.74, *p* = .098.

To examine whether the prevalence of wealth category references in descriptions of another citizen’s life was affected by experimental condition, we ran logistic regression with dummy variables for experimental condition (as above) and target wealth (high wealth = 1, otherwise 0) as predictors for each group in turn. These analyses provided evidence that relative to participants in the low inequality group, those in the high inequality group were more likely to mention Group 2 in Study 3a (low 6% vs. high 19%, *B* = 1.24, odds ratio = 3.44), χ^2^(1) = 7.16, *p* = .007, although not Study 3b (low 14% vs. high 20%, *B* = 0.41, odds ratio = 1.50) χ^2^(1) = 2.32, *p* = .127. Participants in the high inequality group were more likely to mention Group 1 in Study 3a (low 26% vs. high 39%, *B* = 0.83, odds ratio = 2.28), χ^2^(1) = 5.48, *p* = .019, and Study 3b (low 36% vs. high 45%; *B* = 0.80, odds ratio = 2.22), χ^2^(1) = 8.59, *p* = .003. They were also more likely to mention Group 3 in Study 3a (low 24% vs. high 44%, *B* = 1.01, odds ratio = 2.75), χ^2^(1) = 10.68, *p* = .001, and Study 3b (low 33% vs. high 51%, *B* = 0.89, odds ratio = 2.43, χ^2^(1) = 14.38, *p* = .001.

At the same time, when the citizen was said to be a member of Group 1 (rather than Group 3), participants were more likely to reference Group 1 in Study 3a (target poor 7% vs. target rich 69%, *B* = 3.02, odds ratio = 20.43), χ^2^(1) = 50.83, *p* < .001, and Study 3b (target poor 8% vs. target rich 71%; *B* = 3.41, odds ratio = 30.39), χ^2^(1) = 123.68, *p* < .001. They were also more likely to reference Group 2 in Study 3a (target poor 8% vs. target rich 18%, *B* = 0.97, odds ratio = 2.65), χ^2^(1) = 5.05, *p* = .025, but not Study 3b (target poor 14% vs. target rich 20%, *B* = 0.49, odds ratio = 1.62), χ^2^(1) = 3.30, *p* = .069. These participants were also less likely to reference Group 3 in Study 3a (target poor 48% vs. target rich 20%, *B* = −1.39, odds ratio = 0.25), χ^2^(1) = 19.73, *p* < .001, and Study 3b (target poor 66% vs. target rich 19%; *B* = −2.16, odds ratio = 0.12, χ^2^(1) = 82.95, *p* < .001. Analyses that include the gender of the target provide consistent results.

When we examined whether there were any differences in LIWC word frequency as a function of condition, there were a few significant differences. Among other things, when participants in the high inequality condition were describing their own lives, they were significantly less likely to use positive emotion words in Study 3a (low *M* = 5.56, *SD* = 2.93; high *M* = 4.30, *SD* = 2.90), *t*(224) = 3.25, *p* < .001, 95% CI = [0.52, 1.47], *d* = 0.43, and Study 3b (low *M* = 5.42, *SD* = 2.80; high *M* = 4.27, *SD* = 2.37), *t*(414) = 4.51, *p* < .001, 95% CI = [0.65, 1.65], *d* = 0.44. They were also marginally more likely to use negative emotion words in Study 3a (low *M* = 0.77, *SD* = 1.17; high *M* = 1.06, *SD* = 1.27), *t*(224) = 1.80, *p* = .073, 95% CI = [−0.03, 0.61], *d* = 0.24, and significantly more likely to use negative emotion words in Study 3b (low *M* = 0.74, *SD* = 0.93; high *M* = 1.03, *SD* = 1.20), *t*(412) = 2.70, *p* = .007, 95% CI = [0.08, 0.49], *d* = 0.27. (There was little evidence that controlling for positive and negative emotion words accounted for the impact of inequality on wealth references, see SA). Intriguingly, participants in the high inequality condition were significantly more likely to use the third person plural (e.g., “they” or “them”) when describing their own life in Study 3a (low *M* = 0.34, *SD* = 0.70; high *M* = 0.71, *SD* = 0.99), *t*(224) = 3.24, *p* < .001, 95% CI = [0.15, 0.60], *d* = 0.43, and when describing the citizen’s life in Study 3b (low *M* = 0.69, *SD* = 1.12; high *M* = 0.90, *SD* = 1.29), *t*(412) = 1.80, *p* = .076, 95% CI = [−0.02, 0.44], *d* = 0.18. This is consistent with a more intergroup mindset when inequality is higher.

#### Wealth attribute importance

To test H2, we conducted a series of independent samples *t* tests to compare participants’ ratings of the importance of knowing information that was more (vs. less) strongly related to wealth as a function of experimental condition (see Note 2). As expected, we found that in the high (vs. low) inequality condition, participants said that it was more important to know about a stranger’s salary in Study 3a (low *M* = 2.12, *SD* = 1.05; high *M* = 2.48, *SD* = 1.03), *t*(224) = 2.59, *p* = .010, 95% CI = [0.09, 0.63], *d* = 0.34, and in Study 3b (low *M* = 2.13, *SD* = 0.99; high *M* = 2.44, *SD* = 1.14), *t*(412) = 2.92, *p* = .004, 95% CI = [0.10, 0.51], *d* = 0.29. They also said it was more important to know about a stranger’s income group in Study 3a (low *M* = 2.40, *SD* = 1.19; high *M* = 2.85, *SD* = 1.17), *t*(224) = 2.94, *p* = .004, 95% CI = [0.15, 0.75], *d* = 0.39, and Study 3b (low *M* = 2.37, *SD* = 1.16; high *M* = 2.86, *SD* = 1.23), *t*(412) = 4.15, *p* < .001, 95% CI = [0.26, 0.72], *d* = 0.41. In Study 3a, participants in the high (vs. low) inequality condition also said that it was more important to know about a stranger’s occupation (low *M* = 2.97, *SD* = 1.18; high *M* = 3.27, *SD* = 1.00), *t*(224) = 2.07, *p* = .039, 95% CI = [0.01, 0.59], *d* = 0.28, and education (low *M* = 2.83, *SD* = 1.14; high *M* = 3.12, *SD* = 1.02), *t*(224) = 2.05, *p* = .042, 95% CI = [0.01, 0.57], *d* = 0.34. This was not the case in Study 3b: occupation (low *M* = 3.13, *SD* = 1.05; high *M* = 3.16, *SD* = 1.10), *t*(412) = 0.21, *p* = .835, 95% CI = [−0.19, 0.23], *d* = 0.03, and education (low *M* = 2.95, *SD* = 1.09; high *M* = 2.98, *SD* = 1.11), *t*(412) = 0.33, *p* = .742, 95% CI = [−0.18, 0.25], *d* = 0.03.

In Study 3a, there were no significant differences in the perceived importance of the other demographic variables, all *t*(224) ≤ 1.03, all *p* ≥ .305. In Study 3b, participants in the high inequality condition rated information about the stranger’s political view as more important, (low *M* = 2.96, *SD* = 1.16; high *M* = 3.24, *SD* = 1.20), *t*(412) = 2.41, *p* = .016, 95% CI = [0.05, 0.51], *d* = 0.24, but there were no significant differences in the perceived importance of the other demographic variables, all *t*(412) ≤ 1.25, all *p* ≥ .214.

### Discussion

The results of this study generally supported our expectations and the archival results described in Studies 1 and 2. In particular, in line with H1, when participants were asked to imagine living in a more unequal (vs. equal) society, they were more likely to spontaneously reference wealth groups—their own, as well as those of the rich and poor—when describing what life would be like for themselves or another citizen. Furthermore, in line with H2, participants in the more unequal society indicated that it was more important that they know about a stranger’s economic status (in terms of their income group or salary; evidence for occupation and education was weaker) when trying to understand what he or she was like as a person. In sum, then, these findings provide strong evidence of the causal impact of inequality on the salience of wealth. In particular, they support our argument that in societies with more economic inequality, wealth becomes a more fitting lens for seeing the world. The fact that these positive results occurred in the same population—the United States—that had mixed evidence in the archival studies suggests that the cultural factors that affect references to wealth categories in a country’s media may not come into play in a fictional, uncontextualized environment.

In our final study, we aimed to put our findings of the causal impact of inequality perceptions to a much tougher test. Specifically, we aimed to see whether we could replicate our findings when attempting to manipulate participants’ perceptions of economic inequality in their own society. We have argued that *actual* differences in wealth in a given society increase the salience of wealth categories among its members—a claim that has been supported by the preceding studies. Here we investigate whether it is possible to manipulate participants’ *perceptions* of their own society’s inequality and thereby shape their descriptions of what they and others actually do in their daily lives.

## Study 4

### Method

#### Participants

Six hundred thirteen U.K. residents were recruited for a study on wealth and social class in the United Kingdom on the online survey platform *Prolific.co* in exchange for £2.50. One hundred eight participants were excluded according to our preregistered criteria (*N* = 75 did not respond to an open question; *N* = 33 failed attention checks). Therefore, the final sample for analysis purposes consisted of 505 participants. This exceeded our target sample size of 500. This sample was the largest that we could manage within budgetary constraints. We expected that any effects that we did observe would be smaller than those observed in Study 3a and 3b. A sensitivity analysis using G*Power (Faul et al., 2009) revealed that a two-tailed independent samples *t* test with an alpha of .05 would have 80% power to detect a small effect of *d* = 0.25 and 95% power to detect a small-to-medium effect of *d* = 0.32 in this sample.

Participants were 34.26 years old on average, and the majority were female (*N* = 344; male *N* = 157, other responses *N* = 5). More than half of the participants were in part- or full-time employment (full-time *N* = 267; part-time *N* = 104; not employed *N* = 122; retired *N* = 12) and the majority had postsecondary education (*N* = 398). There was a wide spread in self-reported SES, with 30% below average (scores 1–4), 44% about average (scores 5 or 6), and 26% above average (scores 7–10) on the 10-point SES ladder.

#### Procedure

Participants were introduced to the Gini coefficient as a measure of economic inequality and then informed about U.K. performance on this metric. Participants who were randomly allocated to the high (low) inequality condition were told that according to the World Bank, the United Kingdom is more *unequal* on this measure than Slovenia, Hungary, Austria, Croatia, and Korea (more *equal* than Vietnam, Italy, Portugal, Greece, and Lithuania). Participants were then asked to reflect in writing on how this relatively high (low) inequality accords with their own experience of living in the United Kingdom.

After this, in line with the previous studies, participants rated their perceptions of inequality on 7-point Likert-type scales (this scale provided the manipulation check; *r* = .75, *p* < .001), and were asked to provide free response descriptions of their own life in the United Kingdom, as well as that of another U.K. citizen (randomly designated as rich or poor). They were also asked to indicate how important it would be to receive information related to another U.K. resident’s economic status to know what they were like as a person on 5-point Likert-type scales.

### Results

#### Coding

Using the approach described in the previous studies, the first and third authors again independently coded participants’ spontaneous use of wealth categories in their free responses describing their own life and that of another citizen (both were blind to condition). The first coding round resulted in reasonable levels of agreement for rich and poor categories (own life Kappas: 0.52–0.54; citizen life Kappas: 0.49–0.70) but not for the middle class (own life Kappa: 0.52; citizen life Kappa: 0.27). The second coding round produced reasonable to high levels of agreement (own life Kappas: 0.73–0.83; citizen life Kappas: 0.70–0.92). Any remaining disagreements were resolved by the first author.

#### Manipulation check

An independent samples *t* test revealed that participants who read that the United Kingdom had relatively high economic inequality rated the United Kingdom as more unequal than those who read that the United Kingdom had relatively low inequality (low *M* = 5.59, *SD* = 1.01; high *M* = 6.08, *SD* = 0.81), *t*(503) = 6.04, *p <* .001, 95% CI = [0.33, 0.65], *d* = 0.54. Therefore, although both groups of participants perceived the United Kingdom as unequal, this tendency was greater in the high inequality condition.

#### Wealth references

To test H1, we used logistic regressions to examine whether experimental condition (dummy coded: 1 = high inequality, otherwise 0) affected participants’ tendencies to spontaneously reference wealth categories when describing their own life. These analyses revealed that participants who were told that the United Kingdom was a highly unequal society were marginally more likely to reference the poor (low 14% vs. high 20%; *B* = 0.40, odds ratio = 1.49), χ^2^(1) = 2.75, *p* = .097. They were also descriptively more likely to reference the other wealth categories, although these differences were not significant: rich (low 2% vs. high 5%; *B* = 0.68, odds ratio = 1.97), χ^2^(1) = 1.72, *p* = .190; middle class (low 9% vs. high 12%; *B* = 0.27, odds ratio = 1.30), χ^2^(1) = 0.82, *p* = .364. If, however, we examine the tendency to mention *any* wealth group, then we find that participants in the high inequality condition were significantly more likely to reference wealth groups when describing their own life (low inequality 21%, high inequality 29%), χ^2^(1) = 4.11, *p* = .043.

To examine how the manipulation of inequality affected the prevalence of wealth category references in descriptions of another citizen’s life, we again ran logistic regressions with for the inequality manipulation (as above) and target wealth (rich = 1, otherwise 0) as predictors for each wealth category in turn. These analyses revealed that participants who perceived that inequality was higher were significantly more likely to reference the poor (low 36% vs. high 43%; *B* = 0.45, odds ratio = 1.57), χ^2^(1) = 4.33, *p* = .038. They were also descriptively more likely to reference the other groups, although again these differences were not significant: rich (low 34% vs. high 38%; *B* = 0.23, odds ratio = 1.26), χ^2^(1) = 0.81, *p* = .368; middle class (low 10% vs. high 12%; *B* = 0.16, odds ratio = 1.17), χ^2^(1) = 0.29, *p* = .590. If we examine the tendency to mention *any* wealth group, participants in the high inequality condition were significantly more likely to do so (low inequality 64%, high inequality 74%), χ^2^(1) = 5.14, *p* = .023. At the same time, when the citizen was said to be rich (rather than poor), participants were more likely to reference the rich (target poor 2% vs. target rich 69%; *B* = 4.54, odds ratio = 93.88), χ^2^(1) = 108.70, *p* < .001, and the middle class (target poor 6% vs. target rich 16%; *B* = 1.21, odds ratio = 3.34), χ^2^(1) = 13.88, *p* < .001, and less likely to reference the poor (target poor 65% vs. target rich 14%; *B* = −2.45), odds ratio = 0.09, χ^2^(1) = 116.64, *p* < .001.

When we examined whether there were any differences in LIWC word frequency as a function of condition, participants in the low inequality condition were descriptively (but not significantly) more likely to use positive emotion words when describing their own life (low *M* = 4.32, *SD* = 2.46; high *M* = 3.97, *SD* = 2.35), *t*(503) = 1.65, *p* = .101, 95% CI = [−0.07, 0.77], *d* = 0.15; there was no difference in their likelihood to use negative emotion words (low *M* = 1.07, *SD* = 1.34; high *M* = 1.13, *SD* = 1.26), *t*(503) = 0.50, *p* = .617, 95% CI = [−0.29, 0.17], *d* = 0.05.

#### Wealth attribute importance

To test H2, we conducted a series of independent samples *t* tests to compare participants’ ratings of the importance of knowing information that was more (vs. less) strongly related to wealth as a function of experimental condition. Contrary to expectations, participants in the high (vs. low) inequality condition were no more likely to say it was important to know about a stranger’s salary (low *M* = 2.04, *SD* = 0.97; high *M* = 2.03, *SD* = 0.99), *t*(503) = 0.12, *p* = .905, 95% CI = [−0.16, 0.18], *d* = 0.01, social class (low *M* = 2.35, *SD* = 1.05; high *M* = 2.22, *SD* = 1.04), *t*(503) = 1.44, *p* = .150, 95% CI = [−0.05, 0.32], *d* = 0.13, occupation (low *M* = 3.07, *SD* = 1.10; high *M* = 3.04, *SD* = 1.15), *t*(503) = 0.25, *p* = .800, 95% CI = [−0.17, 0.22], *d* = 0.02, or education (low *M* = 2.76, *SD* = 1.12; high *M* = 2.72, *SD* = 1.13), *t*(503) = 0.47, *p* = .640, 95% CI = [−0.15, 0.24], *d* = 0.04. There were also no significant differences in the perceived importance of the other demographic variables, all *t*(503) ≤ 1.37, all *p* ≥ .307.

### Discussion

This study provided partial support for H1. In particular, it revealed that U.K. residents who were led to perceive that there was greater economic inequality in their country were marginally more likely to mention the poor when discussing their own lives and significantly more likely to mention the poor when discussing another citizen’s life. Although we observed the same descriptive pattern when looking at participants’ references to the rich and middle class, these effects were not significant. In contrast, this study did not provide any support for H2, as participants in the high inequality condition did not perceive information about others’ income-related attributes as any more important than those in the low inequality condition. The weaker nature of these findings raises the possibility that the size of the effects in this paradigm was of a “small” size, which our a priori power analysis indicated that we only had a 61% chance of detecting. It is therefore likely that this study was underpowered. These results also suggest that material reality (and existing societal narratives) puts strong constraints on the malleability of perceptions of economic inequality, and that shifting these perceptions in a way that will meaningfully effect people’s tendencies to see their social world through a lens of wealth requires rather more than a simple comparative framing.

## General Discussion

This article provides evidence using a mix of methodological approaches that as economic inequality in a society increases so too does the tendency for people to view society through a lens of wealth. In particular, Study 1 and 2 found that periods in a country’s history that were characterized by greater economic inequality were also characterized by a greater prevalence of wealth category terms like “rich” and “poor” in that country’s books and media publications. Studies 3a and 3b provided evidence that economic inequality plays a causal role in the salience of wealth categories as participants who were asked to imagine a fictional society that was more unequal (vs. equal) were more likely to spontaneous describe that society in terms of wealth and were more concerned about the wealth of other individuals. Finally, Study 4 found that encouraging British participants to perceive the United Kingdom as more economically unequal weakly increased their likelihood of referring to wealth categories when describing their own life and that of other citizens (this did not, however, increase their expressed interest in knowing another person’s wealth).

Together, this work supports Jetten et al.’s (2017) claim that because increasing economic inequality is likely to increase the comparative fit of wealth-based social categories it can be expected to increase the salience of these categories. This effect matters because it is when people see the world in terms of wealth-based social categories that wealth is likely to become an important basis for intergroup dynamics (Turner & Oakes, 1986). In this way, this work contributes to a growing body of social psychological work that aims to inform an understanding of *why* economic inequality appears to be associated with a range of negative social consequences (Jetten & Peters, 2019). In particular, we suggest that one very basic reason that inequality may damage the fabric of society is that it increases the tendency for people to break their social world into the “haves” and the “have nots,” and this feeds into wealth-based intergroup dynamics. At the same time, until the members of unequal societies see their society in these terms, they are likely to do little to address the inequality. Indeed, it is possible that the increased reference to wealth category groups that we observed archivally reflected debates and initiatives that aimed to understand and address rising inequality. In other words, while wealth-based group processes may have negative consequences, they also hold the key to a more equitable future.

Importantly, in showing that the impact of increased inequality on wealth-category salience could be seen in the language that people used to describe the social world—whether in the form of participants’ open responses on a survey, authors’ fictional narratives, or journalists’ factual reports—our article points to the broader social implications of this increased salience. In particular, Studies 1 and 2 suggest that economic inequality may not only shape the way in which individuals see the world, but the way in which they come to construct a shared understanding of the world together (Kashima et al., 2007). And, to the extent that the importance of wealth as a means of parsing the social world becomes a shared part of society’s culture, the more it can be expected to structure collective action seeking to reshape society (Peters & Kashima, 2007; Thomas et al., 2009). In this way, our findings contribute to the broader body of work within the social sciences that has described how the language of social class (not only vocabulary but also dialect and accent) plays an important role in a range of social outcomes, from interpersonal prejudice to class-related politics and policies (e.g., Kinzler, 2021; Tyler, 2008). For instance, Skeggs (2005) argued that the reduction in direct references to social class in the wake of the 1980s reinforced the belief that class was no longer a social problem. More recently, Augoustinos and Callaghan (2019) have analyzed how people’s discussions of income inequality contribute to neoliberal narratives around the importance of individual merit in people’s positions in society. What this work makes clear is that while direct references to wealth groups matter, indirect references to wealth do too, as well as the broader narratives within which these references occur.

The program of work that we present here has a number of strengths, including our use of multiple complementary methods and preregistration (Studies 3b and 4). At the same time, our findings point to some potential constraints to the generalizability of our findings. In particular, while we tested our expectations in a number of countries, for practical reasons, they all had English as a dominant language. The fact that even in this sample we observed some heterogeneity in the association between economic inequality and the prevalence of wealth category references suggests that there are likely to be cultural, historical, and political factors that modulate this association. Potential contenders include perceiver readiness to use wealth-based categories and the normative fit of wealth in a given culture (Turner, 1999; Turner et al., 1987), as well as a culture’s ideological beliefs (e.g., meritocracy). The importance of these factors in shaping people’s responses to economic inequality in everyday life may also account for the fact that in a real economic context (i.e., Study 4), the causal impact of inequality on wealth references was weak and on expressed importance of wealth was nonexistent (this aligns with the findings of the cross-sectional community survey reported in SA). Beyond this, the fact that evidence for H1 (wealth references) was generally stronger than H2 (expressed importance of wealth for social judgments) may indicate that the former measure is more closely tied to category salience and that the latter is more tied to category meaning (i.e., people’s belief that wealth categories are a fair and valid basis for social judgment). If correct, this would speak to the observation that category salience is necessary for negative intergroup dynamics, but is not sufficient for it.

In sum, this work contributes to the growing body of work into the *consequences* of economic inequality by suggesting that one reason it may change the world we live in may be by fundamentally changing the way we see it.

## Research Data

sj-do-1-psp-10.1177_01461672211036627 – Supplemental material for The Language of Inequality: Evidence Economic Inequality Increases Wealth Category Salience Click here for additional data file. Supplemental material, sj-do-1-psp-10.1177_01461672211036627 for The Language of Inequality: Evidence Economic Inequality Increases Wealth Category Salience by Kim Peters, Jolanda Jetten, Porntida Tanjitpiyanond, Zhechen Wang, Frank Mols and Maykel Verkuyten in Personality and Social Psychology Bulletin

sj-do-2-psp-10.1177_01461672211036627 – Supplemental material for The Language of Inequality: Evidence Economic Inequality Increases Wealth Category Salience Click here for additional data file. Supplemental material, sj-do-2-psp-10.1177_01461672211036627 for The Language of Inequality: Evidence Economic Inequality Increases Wealth Category Salience by Kim Peters, Jolanda Jetten, Porntida Tanjitpiyanond, Zhechen Wang, Frank Mols and Maykel Verkuyten in Personality and Social Psychology Bulletin

sj-docx-1-psp-10.1177_01461672211036627 – Supplemental material for The Language of Inequality: Evidence Economic Inequality Increases Wealth Category Salience Click here for additional data file. Supplemental material, sj-docx-1-psp-10.1177_01461672211036627 for The Language of Inequality: Evidence Economic Inequality Increases Wealth Category Salience by Kim Peters, Jolanda Jetten, Porntida Tanjitpiyanond, Zhechen Wang, Frank Mols and Maykel Verkuyten in Personality and Social Psychology Bulletin

sj-docx-2-psp-10.1177_01461672211036627 – Supplemental material for The Language of Inequality: Evidence Economic Inequality Increases Wealth Category Salience Click here for additional data file. Supplemental material, sj-docx-2-psp-10.1177_01461672211036627 for The Language of Inequality: Evidence Economic Inequality Increases Wealth Category Salience by Kim Peters, Jolanda Jetten, Porntida Tanjitpiyanond, Zhechen Wang, Frank Mols and Maykel Verkuyten in Personality and Social Psychology Bulletin

sj-docx-3-psp-10.1177_01461672211036627 – Supplemental material for The Language of Inequality: Evidence Economic Inequality Increases Wealth Category Salience Click here for additional data file. Supplemental material, sj-docx-3-psp-10.1177_01461672211036627 for The Language of Inequality: Evidence Economic Inequality Increases Wealth Category Salience by Kim Peters, Jolanda Jetten, Porntida Tanjitpiyanond, Zhechen Wang, Frank Mols and Maykel Verkuyten in Personality and Social Psychology Bulletin

sj-docx-4-psp-10.1177_01461672211036627 – Supplemental material for The Language of Inequality: Evidence Economic Inequality Increases Wealth Category Salience Click here for additional data file. Supplemental material, sj-docx-4-psp-10.1177_01461672211036627 for The Language of Inequality: Evidence Economic Inequality Increases Wealth Category Salience by Kim Peters, Jolanda Jetten, Porntida Tanjitpiyanond, Zhechen Wang, Frank Mols and Maykel Verkuyten in Personality and Social Psychology Bulletin

sj-docx-5-psp-10.1177_01461672211036627 – Supplemental material for The Language of Inequality: Evidence Economic Inequality Increases Wealth Category Salience Click here for additional data file. Supplemental material, sj-docx-5-psp-10.1177_01461672211036627 for The Language of Inequality: Evidence Economic Inequality Increases Wealth Category Salience by Kim Peters, Jolanda Jetten, Porntida Tanjitpiyanond, Zhechen Wang, Frank Mols and Maykel Verkuyten in Personality and Social Psychology Bulletin

sj-docx-6-psp-10.1177_01461672211036627 – Supplemental material for The Language of Inequality: Evidence Economic Inequality Increases Wealth Category Salience Click here for additional data file. Supplemental material, sj-docx-6-psp-10.1177_01461672211036627 for The Language of Inequality: Evidence Economic Inequality Increases Wealth Category Salience by Kim Peters, Jolanda Jetten, Porntida Tanjitpiyanond, Zhechen Wang, Frank Mols and Maykel Verkuyten in Personality and Social Psychology Bulletin

sj-sav-1-psp-10.1177_01461672211036627 – Supplemental material for The Language of Inequality: Evidence Economic Inequality Increases Wealth Category Salience Click here for additional data file. Supplemental material, sj-sav-1-psp-10.1177_01461672211036627 for The Language of Inequality: Evidence Economic Inequality Increases Wealth Category Salience by Kim Peters, Jolanda Jetten, Porntida Tanjitpiyanond, Zhechen Wang, Frank Mols and Maykel Verkuyten in Personality and Social Psychology Bulletin

sj-sav-2-psp-10.1177_01461672211036627 – Supplemental material for The Language of Inequality: Evidence Economic Inequality Increases Wealth Category Salience Click here for additional data file. Supplemental material, sj-sav-2-psp-10.1177_01461672211036627 for The Language of Inequality: Evidence Economic Inequality Increases Wealth Category Salience by Kim Peters, Jolanda Jetten, Porntida Tanjitpiyanond, Zhechen Wang, Frank Mols and Maykel Verkuyten in Personality and Social Psychology Bulletin

sj-sav-3-psp-10.1177_01461672211036627 – Supplemental material for The Language of Inequality: Evidence Economic Inequality Increases Wealth Category Salience Click here for additional data file. Supplemental material, sj-sav-3-psp-10.1177_01461672211036627 for The Language of Inequality: Evidence Economic Inequality Increases Wealth Category Salience by Kim Peters, Jolanda Jetten, Porntida Tanjitpiyanond, Zhechen Wang, Frank Mols and Maykel Verkuyten in Personality and Social Psychology Bulletin

sj-sps-1-psp-10.1177_01461672211036627 – Supplemental material for The Language of Inequality: Evidence Economic Inequality Increases Wealth Category Salience Click here for additional data file. Supplemental material, sj-sps-1-psp-10.1177_01461672211036627 for The Language of Inequality: Evidence Economic Inequality Increases Wealth Category Salience by Kim Peters, Jolanda Jetten, Porntida Tanjitpiyanond, Zhechen Wang, Frank Mols and Maykel Verkuyten in Personality and Social Psychology Bulletin

sj-sps-2-psp-10.1177_01461672211036627 – Supplemental material for The Language of Inequality: Evidence Economic Inequality Increases Wealth Category Salience Click here for additional data file. Supplemental material, sj-sps-2-psp-10.1177_01461672211036627 for The Language of Inequality: Evidence Economic Inequality Increases Wealth Category Salience by Kim Peters, Jolanda Jetten, Porntida Tanjitpiyanond, Zhechen Wang, Frank Mols and Maykel Verkuyten in Personality and Social Psychology Bulletin

sj-sps-3-psp-10.1177_01461672211036627 – Supplemental material for The Language of Inequality: Evidence Economic Inequality Increases Wealth Category Salience Click here for additional data file. Supplemental material, sj-sps-3-psp-10.1177_01461672211036627 for The Language of Inequality: Evidence Economic Inequality Increases Wealth Category Salience by Kim Peters, Jolanda Jetten, Porntida Tanjitpiyanond, Zhechen Wang, Frank Mols and Maykel Verkuyten in Personality and Social Psychology Bulletin

sj-xlsx-1-psp-10.1177_01461672211036627 – Supplemental material for The Language of Inequality: Evidence Economic Inequality Increases Wealth Category Salience Click here for additional data file. Supplemental material, sj-xlsx-1-psp-10.1177_01461672211036627 for The Language of Inequality: Evidence Economic Inequality Increases Wealth Category Salience by Kim Peters, Jolanda Jetten, Porntida Tanjitpiyanond, Zhechen Wang, Frank Mols and Maykel Verkuyten in Personality and Social Psychology Bulletin

sj-xlsx-2-psp-10.1177_01461672211036627 – Supplemental material for The Language of Inequality: Evidence Economic Inequality Increases Wealth Category Salience Click here for additional data file. Supplemental material, sj-xlsx-2-psp-10.1177_01461672211036627 for The Language of Inequality: Evidence Economic Inequality Increases Wealth Category Salience by Kim Peters, Jolanda Jetten, Porntida Tanjitpiyanond, Zhechen Wang, Frank Mols and Maykel Verkuyten in Personality and Social Psychology Bulletin

sj-xlsx-3-psp-10.1177_01461672211036627 – Supplemental material for The Language of Inequality: Evidence Economic Inequality Increases Wealth Category Salience Click here for additional data file. Supplemental material, sj-xlsx-3-psp-10.1177_01461672211036627 for The Language of Inequality: Evidence Economic Inequality Increases Wealth Category Salience by Kim Peters, Jolanda Jetten, Porntida Tanjitpiyanond, Zhechen Wang, Frank Mols and Maykel Verkuyten in Personality and Social Psychology Bulletin

sj-xlsx-4-psp-10.1177_01461672211036627 – Supplemental material for The Language of Inequality: Evidence Economic Inequality Increases Wealth Category Salience Click here for additional data file. Supplemental material, sj-xlsx-4-psp-10.1177_01461672211036627 for The Language of Inequality: Evidence Economic Inequality Increases Wealth Category Salience by Kim Peters, Jolanda Jetten, Porntida Tanjitpiyanond, Zhechen Wang, Frank Mols and Maykel Verkuyten in Personality and Social Psychology Bulletin

sj-xlsx-5-psp-10.1177_01461672211036627 – Supplemental material for The Language of Inequality: Evidence Economic Inequality Increases Wealth Category Salience Click here for additional data file. Supplemental material, sj-xlsx-5-psp-10.1177_01461672211036627 for The Language of Inequality: Evidence Economic Inequality Increases Wealth Category Salience by Kim Peters, Jolanda Jetten, Porntida Tanjitpiyanond, Zhechen Wang, Frank Mols and Maykel Verkuyten in Personality and Social Psychology Bulletin

sj-xlsx-6-psp-10.1177_01461672211036627 – Supplemental material for The Language of Inequality: Evidence Economic Inequality Increases Wealth Category Salience Click here for additional data file. Supplemental material, sj-xlsx-6-psp-10.1177_01461672211036627 for The Language of Inequality: Evidence Economic Inequality Increases Wealth Category Salience by Kim Peters, Jolanda Jetten, Porntida Tanjitpiyanond, Zhechen Wang, Frank Mols and Maykel Verkuyten in Personality and Social Psychology Bulletin

sj-xlsx-7-psp-10.1177_01461672211036627 – Supplemental material for The Language of Inequality: Evidence Economic Inequality Increases Wealth Category Salience Click here for additional data file. Supplemental material, sj-xlsx-7-psp-10.1177_01461672211036627 for The Language of Inequality: Evidence Economic Inequality Increases Wealth Category Salience by Kim Peters, Jolanda Jetten, Porntida Tanjitpiyanond, Zhechen Wang, Frank Mols and Maykel Verkuyten in Personality and Social Psychology Bulletin

## Notes

1 While this rate of exclusions may appear high, there is evidence that this may not be unusual among researchers using MTurk participants for studies that include free text responses. Specifically, in their blog describing replications of consumer psychology studies with MTurk participants (http://datacolada.org/archives/category/data-replicada), Nelson and Simmons report dropout rates of 22% to 42% for studies that include an open response writing task; when they considered the quality of the responses, they excluded another 17% of the sample (http://datacolada.org/89). Further discussion of the exclusion rate and related analysis is available in Supplemental Analyses (SA).

2 With the Bonferroni correction, only income group differs significantly in Studies 3a and 3b.
