# Supplementary material for: The Language of Inequality: Evidence Economic Inequality Increases Wealth Category Salience
Source: Pers Soc Psychol Bull. 2021 Aug 5;48(8):1204–19. doi: 10.1177/01461672211036627 (PMC9245161; doi:10.1177/01461672211036627)
Supplement: sj-docx-1-psp-10.1177_01461672211036627 – Supplemental material for The Language of Inequality: Evidence Economic Inequality Increases Wealth Category Salience [file sj-docx-1-psp-10.1177_01461672211036627.docx]

LIWC Dictionary

%
1 group1
2 group2
3 group3

%
1* 1
1st class 1

1st group 1

1st tier 1

1st level 1

Above me 1

Above us 1

Advantag* 1

Affluent 1

Better off 1

Category 1 1

Category one 1

Category a 1

Class 1 1

Class one 1

Different economic 1

Different social 1

Elite 1

Fancy 1

First class 1

First group 1

First tier 1

Group 1 1

Group one 1

Group above 1

Group-1 1

High class* 1

High group 1

High income 1

High level 1

High position 1

High social 1

High socio* 1

High status 1

Higher class* 1

Higher level 1

Higher group 1

Higher income 1

Higher position 1

Higher social 1

Higher socio* 1

Higher status 1

Highest class 1

Highest level 1

Highest group 1

Highest income 1

Highest position 1

Highest social 1

Highest socio* 1

High salary 1

Higher salary 1

Income 1 1

Leisure class 1

Level 1 1

Level one 1

luxur* 1

Mega-rich 1

Megarich 1

More fortunate 1

Other class* 1

Other group* 1

Other status 1

Prestig* 1

Rich* 1

Super-rich 1

Snob* 1

Top class* 1

Top group 1

Top income 1

Top level 1

Top position 1

Top social 1

Top tier 1

Uber-rich 1

Upper class* 1

Upper income 1

Wealth* 1

2* 2

2nd tier 2

2 class* 2

2nd class* 2

2nd level 2

Average class 2

Average income 2

Average level 2

Average salary 2

Average people 2

Average position 2

Average social 2

Average socio* 2

Average status 2

Average-class 2

Category 2 2

Category B 2

Class 2 2

Group 2 2

Group two 2

Group-2 2

Income 2 2

Level 2 2

Middle class* 2

Middle group 2

Middle income 2

Middle level 2

Middle position 2

Middle salary 2

Middle social 2

Middle socio* 2

Middle status 2

Middle-class 2

Mid class* 2

Mid level 2

Mid salary 2

Mid status 2

My class 2

My group 2

My income 2

My level 2

My place 2

My position 2

My social 2

My status 2

Normal income 2

Own class 2

Own group 2

Own income 2

Own kind 2

Own level 2

Own place 2

Own position 2

Own social 2

Own status 2

People like me 2

Same class 2

Same economic 2

Same group 2

Same level 2

Same lifestyle 2

Same position 2

Same social 2

Same socioeconomic 2

Same status 2

Second class 2

Second group 2

Second income 2

Second tier 2

Similar class 2

Similar Group 2

Similar income 2

Similar lives 2

Similar position 2

Similar situation 2

Similar status 2

3* 3

3rd class 3

3rd group 3

3rd tier 3

3rd level 3

Below me 3

Below us 3

Bottom class 3

Bottom group 3

Bottom level 3

Bottom position 3

Bottom social 3

Bottom status 3

Category 3 3

Category c 3

Category three 3

Class 3 3

Class three 3

Different economic 3

Different social 3

Disadvantag* 3

Group 3 3

Group below 3

Group three 3

Income 3 3

Less fortunate 3

Level 3 3

Level three 3

Low class* 3

Low income 3

Low level 3

Low position 3

Low salary 3

Low social 3

Low socio* 3

Low status 3

Lower class 3

Lower class* 3

Lower income 3

Lower level 3

Lower position 3

Lower salary 3

Lower social 3

Lower socio* 3

Lower status 3

Lower-income 3

Lowest class* 3

Lowest group 3

Lowest income 3

Lowest level 3

Lowest position 3

Lowest salary 3

Lowest social 3

Lowest socio* 3

Lowest status 3

Other class* 3

Other group* 3

Other status 3

Poor* 3

Poverty* 3

Riff raff 3

Third class 3

Third group 3

Third tier 3

Worse off 3

Worst off 3
