# Supplementary material for: The Language of Inequality: Evidence Economic Inequality Increases Wealth Category Salience
Source: Pers Soc Psychol Bull. 2021 Aug 5;48(8):1204–19. doi: 10.1177/01461672211036627 (PMC9245161; doi:10.1177/01461672211036627)
Supplement: sj-docx-3-psp-10.1177_01461672211036627 – Supplemental material for The Language of Inequality: Evidence Economic Inequality Increases Wealth Category Salience [file sj-docx-3-psp-10.1177_01461672211036627.docx]

**Introduction**

Welcome to this study!

Participant Information Sheet

The purpose of this study is to examine your experience living in a fictional society. This study is being conducted by

Participation and Withdrawal

Participation in this study is completely voluntary and you are free to withdraw at any time without any penalty. If you wish to withdraw, simply stop completing the survey. If you do choose to withdraw from the study, all materials that you have completed will be deleted and will not be included in the study.

What is Involved?

Participants will be asked to respond to several questions that assess their perception, preference and memory. Participation in this study will take 10-20 minutes.

Payment

We value participants who give our study their full attention. To ascertain whether or not you are paying attention, we include 3 comprehension checks in our survey. Participants who pass these comprehension checks will be able to complete the entire study and will be paid USD 2.50 for taking part. Participants who do not pass these comprehension checks will not be able to complete the entire survey and will not be paid (also please return the HIT on MTurk so that your approval rating is not affected). **For MTurk workers, if you would like to receive your reimbursement, once you complete the checks, please complete the survey to receive the code you will need for payment.**

Risks

Participation in this study should involve no physical or mental discomfort, and no risks beyond those of everyday living. If, however, you find any question or procedure to be uncomfortable or offensive, you are free to omit from answering or participating in that aspect of the study.

Confidentiality and Security of Data

All data collected in this study will be stored confidentially. Only members of the research team will have access to the data. All data will be coded in a de-identified manner and subsequently analysed and reported in such a way that responses will not be able to be linked to any individual. The data you provide will only be used for the research purpose of this study.

Ethics Clearance and Contacts

This study has been cleared in accordance with the ethical review processes of the University of

If you would like to learn the outcome of the study in which you are participating, you can contact me using the email above and I will send you an Abstract of the study and findings.

Thank you for your participation in this study.

**Clicking the 'Next' button confirms that you have read and understood the information provided above, and decided to participate as a research subject for this study.**

**Main comprehension check instruction**

In this study, we are interested in people's perceptions and opinions in a fictitious social context. You will become a citizen of a new society called Bimboola. Imagine that you will start a new life there, and become a member of Bimboolean society.

Bimboola is just like any other society that consists of a few social groups. There are **3 main income groups** in Bimboolean society.

In order to start off your new life in Bimboola, you will **be randomly assigned to one of these income levels**.

After the assignment, you will be able to **choose a house, a car, and a trip** to start your life in Bimboola. Then you will be asked to respond to a series of questions asking you what you think of the Bimboola society. At the end of the study, you will be asked several questions concerning your demographics and opinions in the real world.

When you are confident that you understand what this study involves, please input your MTurk ID (which is required for reimbursement) and click on the "Next" button.

MTurk ID

# Comprehension checks

In order to complete this study, it is important that you understand how the study works. Please answer these three comprehension checks. If you answer them correctly, you will be invited to complete the study.

How many income groups are there in Bimboolean society?

1 2 3 4 5

In order to start your new life in Bimboola, you will be randomly assigned to an income group.

True False

After you have been assigned to an income group, you will be asked to choose 4 things (a house, car, trip and job).

True False

**Failed attention checks**

You've answered the question incorrectly and cannot proceed on with the survey. We thank you for your participation. Have a great day!

**Inequality Condition Structure**

**Bimboola**

In this study you will become a citizen of a new society called Bimboola. You will start a new life there, and become a member of the Bimboolean society.

**Bimboola is a society that is quite unequal in its wealth distribution. This means that the wealth gap between the poorest and wealthiest people in Bimboola is**

**quite large (in particular when comparing it to other societies).**

In Bimboola, **wealth is organized in three levels** depending on mean levels of income. The income distribution takes the shape of the picture below.


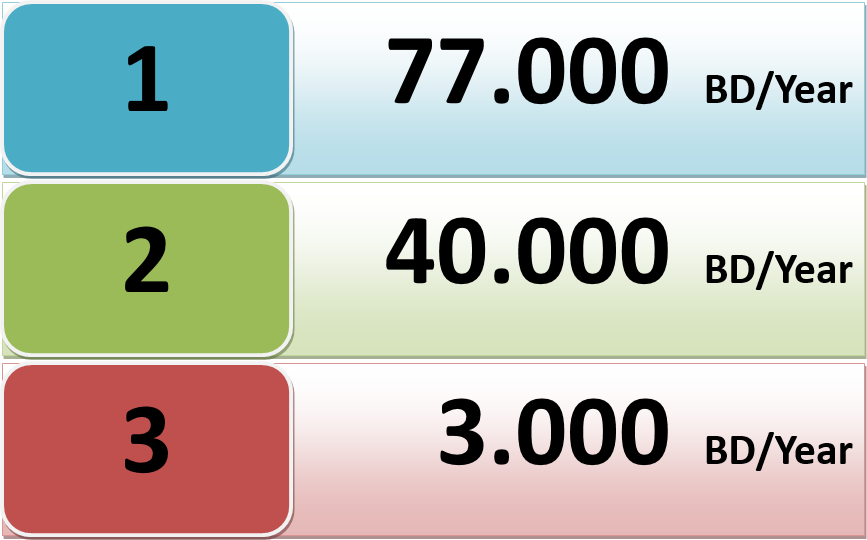


You can see in the picture how much people in each income group earn on average:

- **income group 1** earns on average 77.000 BD per year
- **income group 2** earns on average 40.000 BD per year
- **income group 3** earns on average 3.000 BD per year

In order to start off your new life in Bimboola you are going to be assigned to one of these income levels.

On the next page, you will receive information about the income group to which you have been assigned to. Please read this information carefully.

**Equality Condition Structure**

**Bimboola**

In this study you will become a citizen of a new society called Bimboola. You will start a new life there, and become a member of the Bimboolean society.

**Bimboola is a society that is quite equal in its wealth distribution. This means that the wealth gap between the poorest and wealthiest people in Bimboola is quite small (in particular when comparing it to other societies).**

In Bimboola, **wealth is organized in three levels** depending on mean levels of income. The income distribution takes the shape of the picture below.


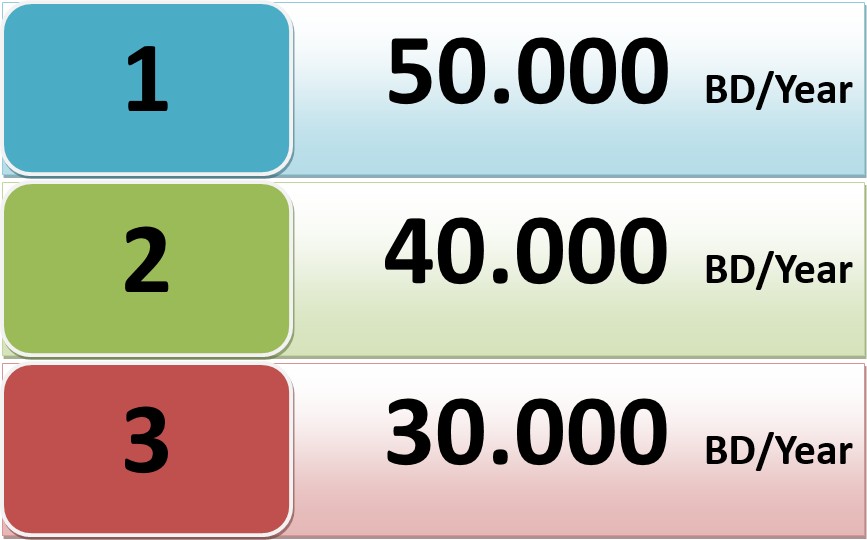


You can see in the picture how much people in each income group earn on average:

- **income group 1** earns on average 50.000 BD per year
- **income group 2** earns on average 40.000 BD per year
- **income group 3** earns on average 30.000 BD per year

In order to start off your new life in Bimboola you are going to be assigned to one of

these income levels.

On the next page, you will receive information about the income group to which you have been assigned to. Please read this information carefully.

**IC Assigned**

You have been assigned to **income group 2**


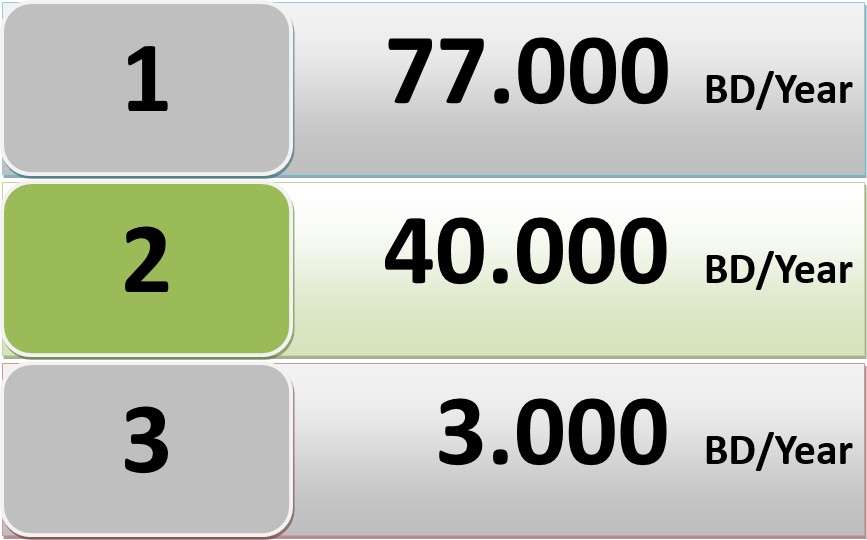


Bimboolean citizens in **income group 2** earn on average 40.000 BD/Year

**Comprehension check - income group**

Which income group have you been assigned to?

Income group 1

Income group 2

Income group 3

**New Life**

To begin your new life in Bimboola, the daily essentials must be purchased. The following questions will ask you to select items that you need to live your life in Bimboola.

PLEASE NOTE: You can only select the items that you can afford as governed by your income group. You are assigned to **income group 2**, so you can only afford to select items from category 2 or 3.

**Houses Introd**

In the next slides you will see different houses available in Bimboola. The houses are grouped into 3 different categories depending on their prices. For instance, the prices of houses in category 1 (i.e.,1a, 1b, 1c) can only be afforded by people from income group 1.

Firstly, you will see houses that people from different income groups can afford. After viewing these options, you will later be asked to select your house.

IMPORTANT: You can only choose from those houses that your income group can afford. The items are numbered by income group so you know what you can afford.

**IC Houses 1**

Below are houses that people from **income group 1** can afford.

1A


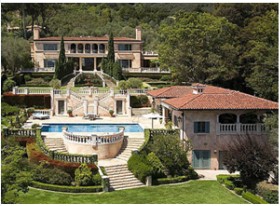


1B


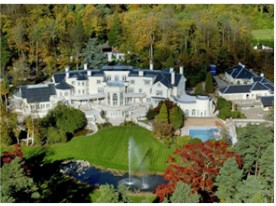


1C


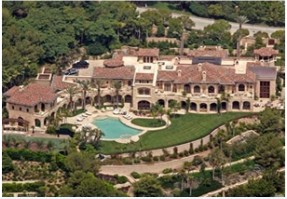


**Houses2**

Below are houses that people in **income group 2** can afford.

2A


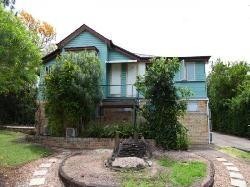


2B


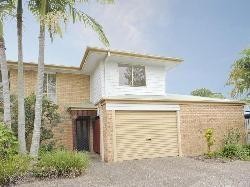


2C


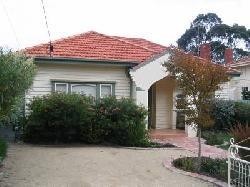


**IC Houses 3**

Below are houses that people from **income group 3** can afford.

3A


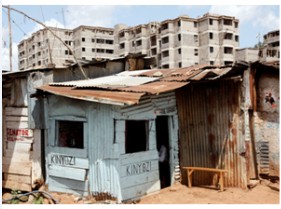


3B


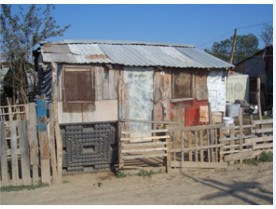


3C


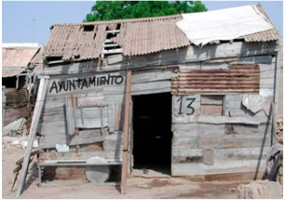


**IC Houses Choose**

Please select the house you would like to buy by clicking on one of the pictures:


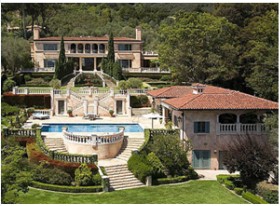
Remember you can only choose from those houses that your income group can afford. You belong to **income group 2**, so you can only afford to select houses from category 2 or 3.


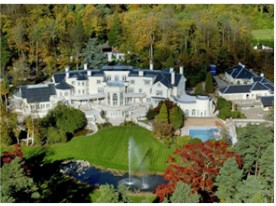
1A

1B

1C
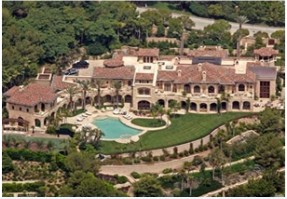


2A
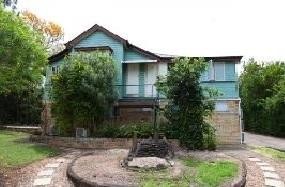


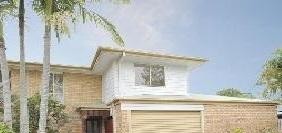


**Cars Introd**

# You've selected your house!

Now you will need to select your means of transport to get around in Bimboola.

In the next slides you will see different means of transport available in Bimboola. They are organized in three groups of three means of transport depending on

their prices. For instance, the prices of the types of transport in category 1 (i.e.,1a, 1b, 1c) can only be afforded by people from income group 1.

Firstly, you will see the transportation types that people from different income groups can afford. After viewing these options, you will later be asked to select your transportation.

IMPORTANT: You can only choose the means of transport that your income group can afford. The items are numbered by income group so you know what you can afford.

**IC Cars1**

Below are transportation types that people from **income group 1** can afford.

1A


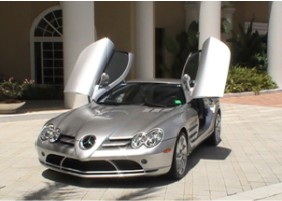


1B


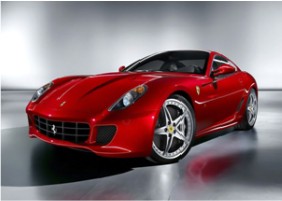


1C


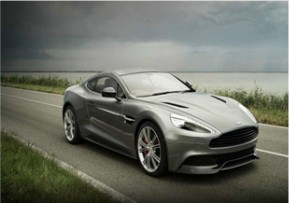


**Cars2**

Below are transportation types that people in **income group 2** can afford.

2A


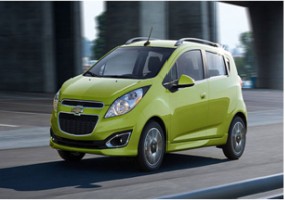


2B


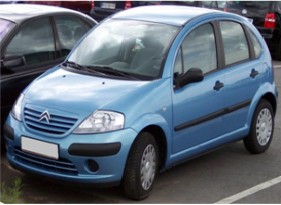


2C


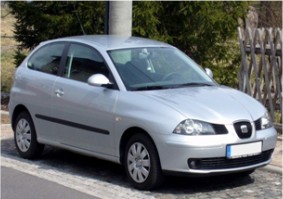


**IC Cars3**

Below are transportation types that people from **income group 3** can afford.

3A


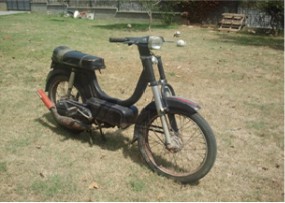


3B


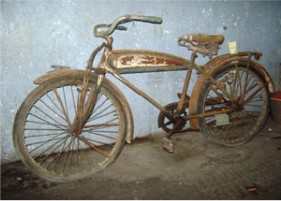


3C


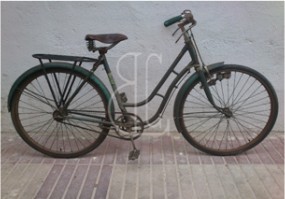


**IC Cars Choose**

Please select the means of transport you would like to buy by clicking on one of the pictures:

Remember you can only choose from those means of transport that your income group can afford. You belong to **income group 2**, so you can only afford to select the type of transport from category 2 or 3.


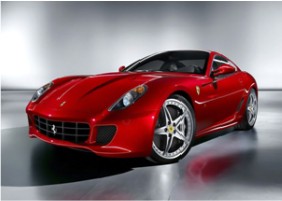
1A
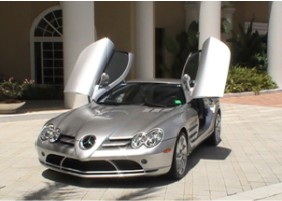


1B

1C
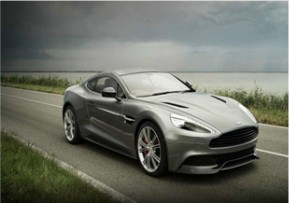


2A
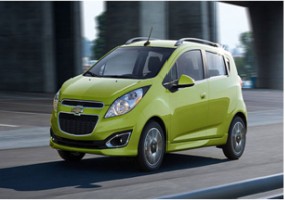


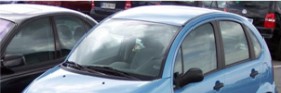


**Holidays Introd**

# You've selected your means of transport!

Now you will need to select the type of holidays you would like to go on.

In the next slides you will see different holidays available in Bimboola. They are organized in three groups of three holidays depending on their prices. For instance, the prices of the holidays in category 1 (i.e.,1a, 1b, 1c) can only be afforded by people from income group 1.

Firstly, you will see the holidays that people from different income groups can afford. After viewing these options, you will later be asked to select your holiday.

IMPORTANT: You can only choose the holiday that your income group can afford. The items are numbered by income group so you know what you can afford.

**IC Holidays1**

Below are holidays that people from **income group 1** can afford.

1A- A space trip (a day trip in a space shuttle)


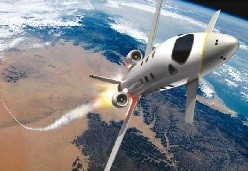


1B- Holidays on a private island (an overseas trip, as long as you like)


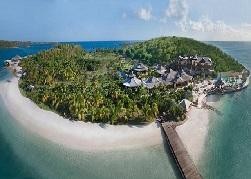


1C- Luxury hotel holiday in an exotic country (an overseas trip, as long as you like)


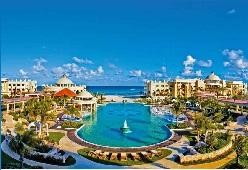


**Holidays2**

Below are holidays that people in **income group 2** can afford.

2A- A motorhome holiday (domestic, 1 week)


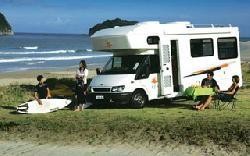


2B- A 4WD adventure (domestic, 1 week)


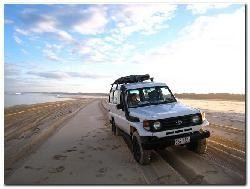


2C- A stay at a holiday village (domestic, 1 week)


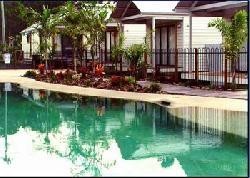


**IC Holidays3**

**Unfortunately, people in income group 3 cannot afford a holiday.**

**IC Holidays Choose**

### Please select the holiday type that you would like to enjoy by clicking on one of the pictures:

Remember you can only choose the holiday that your income group can afford. You belong to **income group 2**, so you can only afford to select the holiday from category 2 or 3.


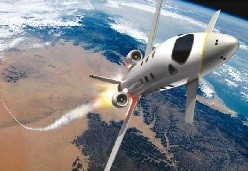
1A- A space trip (day trip in a space shuttle)

1B- Holidays in your private island (overseas, as long as you like)


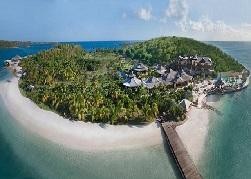


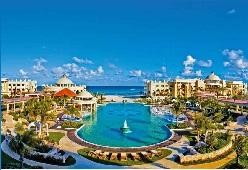
1C- Luxury hotel in an exotic place (overseas, as long as you like)

2A - A motorhome holiday (domestic, 1 week)


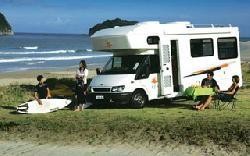


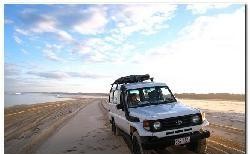
2B - A 4WD adventure (domestic, 1 week)

**EC Assigned**

You have been assigned to **income group 2**


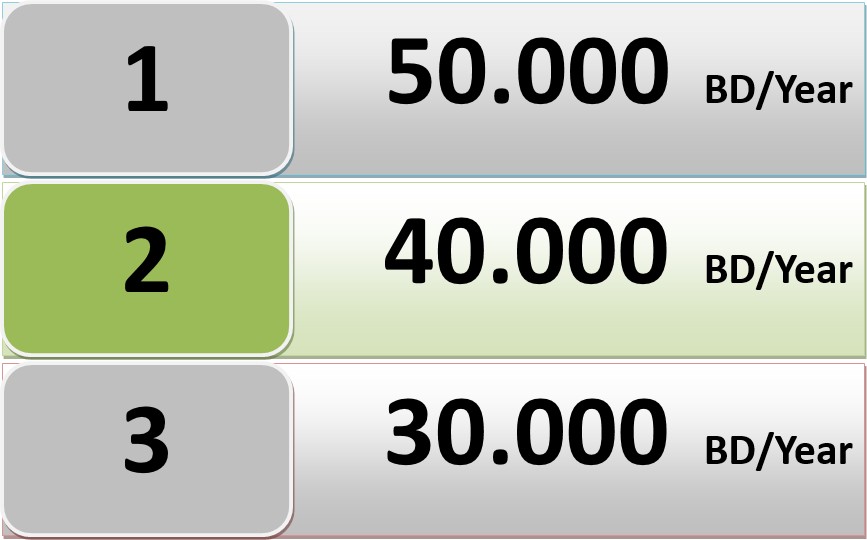


Bimboolean citizens in **income group 2** earn on average 40.000 BD/Year

**EC Houses 1**

Below are houses that people from **income group 1** can afford.

1A


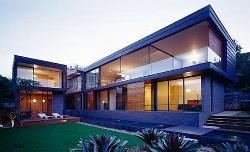


1B


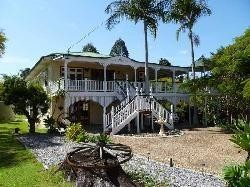


1C


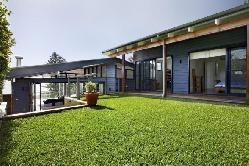


**EC Houses 3**

Below are houses that people from **income group 3** can afford.

3A


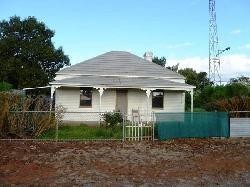


3B


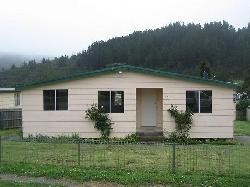


3C


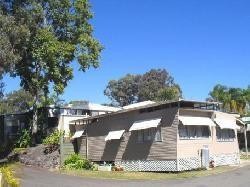


**EC Houses Choose**

Please select the house you would like to buy by clicking on one of the pictures:

Remember you can only choose from those houses that your income group can afford. You belong to **income group 2**, so you can only afford to select houses from category 2 or 3.

1A
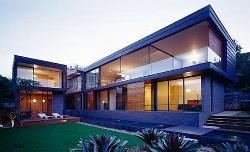


1B
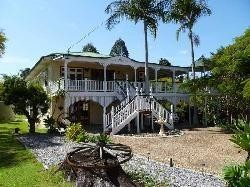


1C
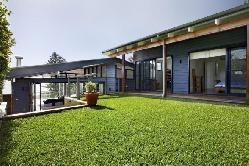


2A
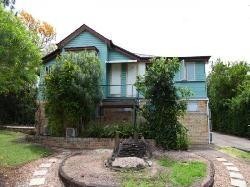


2B
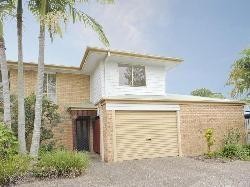


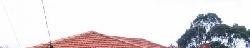


**EC Cars1**

Below are transportation types that people from **income group 1** can afford.

1A


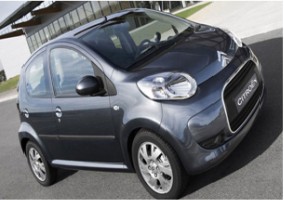


1B


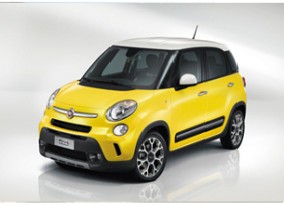


1C


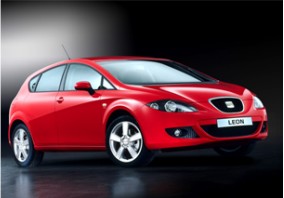


**EC Cars3**

Below are transportation types that people from **income group 3** can afford.

3A


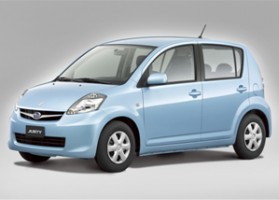


3B


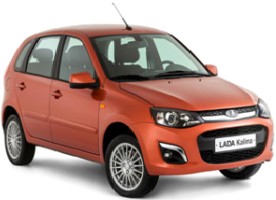


3C


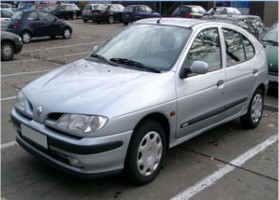


**EC Cars Choose**

### Please select the means of transport you would like to buy by clicking on one of the pictures:

Remember you can only choose from those means of transport that your income group can afford. You belong to **income group 2**, so you can only afford to select the type of transport from category 2 or 3.

1A
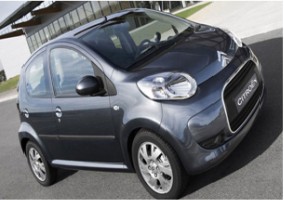


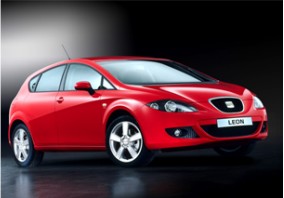
1B
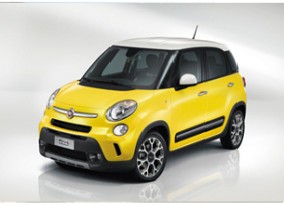


1C

2A
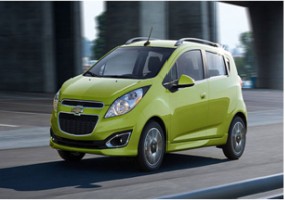


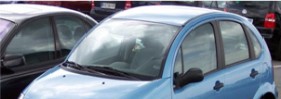


**EC Holidays1**

Below are holidays that people from **income group 1** can afford.

1A - City hopping in Europe (overseas, 2 weeks)


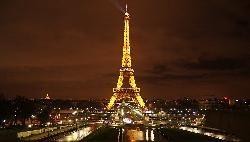


1B - A cultural trip to Thailand (overseas, 2 weeks)


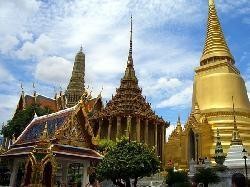


1C - A snorkeling-diving holiday (domestic, 2 weeks)


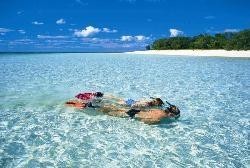


**EC Holidays3**

Below are holidays that people from **income group 3** can afford.

3A - A weekend at the beach (domestic)


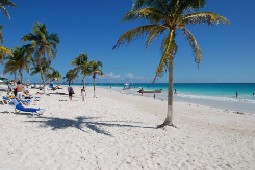


3B - A camping trip (domestic, 3 days)


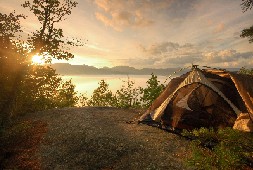


3C - A weekend at a theme park (domestic)


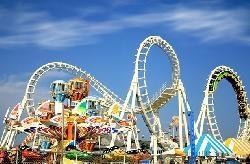


**EC Holidays Choose**

### Please select the holiday type that you would like to enjoy by clicking on one of the pictures:

Remember you can only choose the holiday that your income group can afford. You belong to **income group 2**, so you can only afford to select the holiday from category 2 or 3.


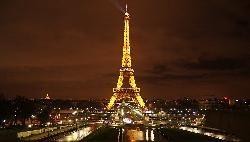
1A - City hopping in Europe (overseas, 2 weeks)

1B - A cultural trip to Thailand (overseas, 2 weeks)


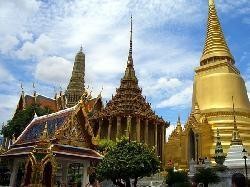


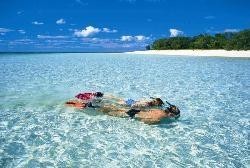
1C - A snorkeling-diving holiday (domestic, 2 weeks)

2A - A motorhome holiday (domestic, 1 week)


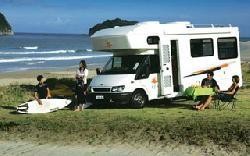


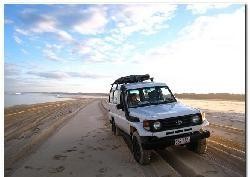
2B - A 4WD adventure (domestic, 1 week)

**Manipulation Check, Wealth and Identity**

**You've selected your holiday!**

Now that you've purchased the essential items that you need to live in Bimboola, please answer the following questions based on your perception of Bimboola society:

Which income group have you been assigned to?

| 1  How wealthy is your group? |  | 2 |  |  | 3 |  |
| --- | --- | --- | --- | --- | --- | --- |
| 1 (Very 2 3  Poor) | 4 | 5 (Average) | 6 | 7 | 8 | 9 (Very Wealthy) |

How poor is your group?

| 1 (Very | 2 | 3 | 4 | 5 (Average) | 6 | 7 | 8 | 9 (Very |
| --- | --- | --- | --- | --- | --- | --- | --- | --- |
| Poor) |  |  |  |  |  |  |  | Wealthy) |

Consider the wealthiest group in Bimboola (i.e., income group 1), how wealthy is this group?

| 1 (Not at | 2 | 3 | 4 | 5 (Average) | 6 | 7 | 8 | 9 (Very |
| --- | --- | --- | --- | --- | --- | --- | --- | --- |
| all Wealthy) |  |  |  |  |  |  |  | Wealthy) |

Consider the poorest group in Bimboola (i.e., income group 3), how wealthy is this group?

| 1 (Not at | 2 | 3 | 4 | 5 (Average) | 6 | 7 | 8 | 9 (Very |
| --- | --- | --- | --- | --- | --- | --- | --- | --- |
| all Wealthy) |  |  |  |  |  |  |  | Wealthy) |

How unequal is Bimboola?

| 1 (Not at | 2 | 3 | 4 | 5 (Average) | 6 | 7 | 8 | 9 (Very |
| --- | --- | --- | --- | --- | --- | --- | --- | --- |
| all) |  |  |  |  |  |  |  | much) |

How equal is Bimboola?

| 1 (Not at | 2 | 3 | 4 | 5 (Average) | 6 | 7 | 8 | 9 (Very |
| --- | --- | --- | --- | --- | --- | --- | --- | --- |
| all) |  |  |  |  |  |  |  | much) |

To what extent do you identify as a Bimboolean?

1 (Not at all) 2 3 4 (Neutral) 5 6 7 (Very much)

To what extent do you identify as a member of income group 2?

1 (Not at all) 2 3 4 (Neutral) 5 6 7 (Very much)

**Intro Description Task**

Now that you have the essential items to start off your life in Bimboola society, imagine your life as a Bimboolean. **In 150-200 words, describe what life is like for you.**

Generally, describe how your normal day would unfold. You can talk about: 1.) Your ***daily activities*** (i.e., outline your plans for the day).

2.) Your main ***social interactions*** (i.e., the individuals you would meet,

their relationships to you, how you would interact with them and the tone of the interaction).

3.) Your ***thoughts and feelings*** throughout your day in Bimboola (i.e., how you would feel waking up, going about your daily activities and meeting the individuals you have described).

**Intro to Bimboolean Christine**

## Imagine that one day you meet another Bimboolean citizen named

Christine. Christine has lived in Bimboola all of her life.


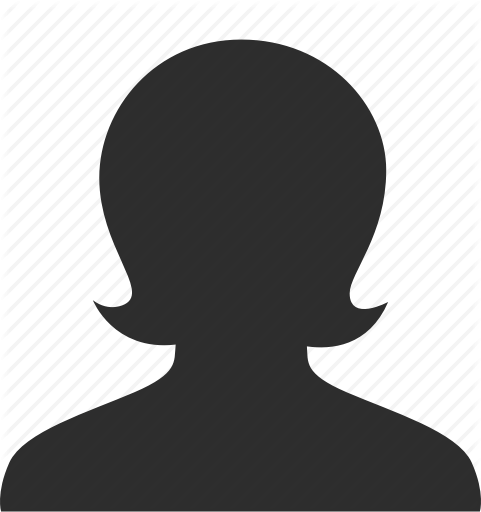


**Intro to Bimboolean Chris M**

## Imagine that one day you meet another Bimboolean citizen named Chris. Chris has lived in Bimboola all of his life.


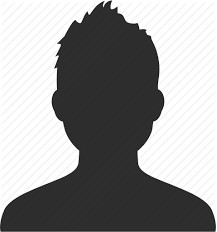


**Christine_Getting to know task**

Now imagine that you want to get an idea of what Christine is like as a person.

There are, of course, many pieces of information that you could use to get a better idea of what Christine is like. We have provided a list of potential pieces of information below. You may feel that some of these pieces of information are *very important* for getting to know what Christine is like, and some other information are *not at all important* in forming a judgement of Christine.

For each of the information, please indicate how important you think it is for knowing what Christine is like.

**"It is very important to me that I know if Christine is..."**

Strongly No No Neutral Yes

Strongly Yes

Righteous
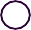

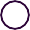

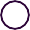

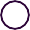

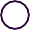


Honest
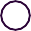

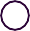

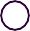

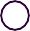

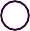


Sincere
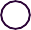

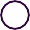

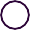

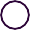

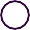


Respectful
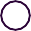

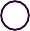

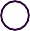

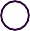

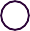


Trustworthy
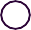


**"It is very important to me that I know if Christine is..."**

Strongly No No Neutral Yes

Strongly Yes

Friendly

Likeable

Kind

Warm

Helpful

**"It is very important to me that I know if Christine is..."**

Strongly No No Neutral Yes

Strongly Yes

Intelligent

Competent

Skilful

Eﬃcient

Capable

**"It is very important to me that I know Christine's.."**

Strongly No No Neutral Yes

Strongly Yes

Age

Ethnicity

Occupation

Salary

Religion

Political view

Income group

Education

Hobby

**"It is very important to me that I know that Christine is..."**

|  | Strongly No | No | Neutral | Yes | Strongly Yes |
| --- | --- | --- | --- | --- | --- |
| A caring person, who cherishes and protects others |  |  |  |  |  |
| A fair person, who will never cheat or break the rules |  |  |  |  |  |
| A loyal Bimboolean, who will stand with other Bimbooelans |  |  |  |  |  |
| A respectful person, who will uphold traditions and obey authority |  |  |  |  |  |
| A person who is physically and spiritually clear and pure |  |  |  |  |  |
| A ... This is a control question, please select "Strongly No" |  |  |  |  |  |

**Chris_Getting to know task**

Now imagine that you want to get an idea of what Chris is like as a person.

There are, of course, many pieces of information that you could use to get a better idea of what Chris is like. We have provided a list of potential pieces of information below. You may feel that some of these pieces of information are *very important* for getting to know what Chris is like, and some other information are *not at all important* in forming a judgement of Chris.

For each of the information, please indicate how important you think it is for knowing what Chris is like.

**"It is very important to me that I know if Chris is.."**

Strongly No No Neutral Yes

Strongly Yes

Righteous

Trustworthy

Sincere

Honest

Respectful

**"It is very important to me that I know if Chris is.."**

Strongly No No Neutral Yes

Strongly Yes

Likeable

Friendly

Kind

Helpful

Warm

**"It is very important to me that I know if Chris is.."**

Strongly No No Neutral Yes

Strongly Yes

Competent

Intelligent

Capable

Eﬃcient

Skilful

**"It is very important to me that I know Chris's..."**

Strongly No No Neutral Yes

Strongly Yes

Hobby

Income group

Salary

Religion

Age

Education

Ethnicity

Occupation

Political view

**"It is very important to me that I know that Chris is..."**

|  | Strongly No | No | Neutral | Yes | Strongly Yes |
| --- | --- | --- | --- | --- | --- |
| A caring person, who cherishes and protects others |  |  |  |  |  |
| A fair person, who will never cheat or break the rules |  |  |  |  |  |
| A loyal Bimboolean, who will stand with other Bimbooleans |  |  |  |  |  |
| A respectful person, who will uphold traditions and obey authority |  |  |  |  |  |
| A person who is physically and spiritually clear and pure |  |  |  |  |  |
| A ... This is a control question, please select "Strongly No" |  |  |  |  |  |

**Jane_Rich_Statements**

In your daily life at Bimboola, you meet **another Bimboolean** called Jane. Jane has lived in Bimboola all of her life.

Below are statements that will help you form an impression about Jane. Please take your time to carefully look through the statements. In the next section, you will be asked to describe Jane and your impression of her.

Jane works at an advertising company.

Jane was married but recently divorced.

Jane owns an expensive sports car.

On weekends, Jane visits her parents.

Jane has a dog.

Jane is not a morning person.

Jane just booked a trip to go on a five-stared cruise.

Jane likes to go to the gym after work.

Jane likes to eat cereal for breakfast.

Jane rides a bicycle to work.

Jane’s father is a neurosurgeon.

Jane has two children.

Jane's children go to a prestigious private school.

Jane always travel first class on airplanes.

Jane likes going to musicals.

Jane is from income group 1.

Jane enjoys public speaking.

Jane doesn’t like to eat broccoli.

Jane likes to eat rice.

Jane can speak two languages.

Jane's favourite class in high school was math.

**Jane_Statements_Description task**

From what you know about Jane, how do you think she is as a person? **In 150-200 words, please describe your impression of her**. You can focus on the following:

1. What do you think is her usual daily routine? (e.g., her plan for a normal day)
2. Who do you think she usually interacts with? (e.g., her family, her colleagues)
3. What do you think would be her general mood and temperament? (e.g., is she an optimistic/a pessimistic person)

To what extent do you like or dislike Jane as a person?

- 1. (I dislike Jane very much)
  2. 3 4 5 6 7 (I like Jane

very much)

**Jane_Poor_Statements**

In your daily life at Bimboola, you meet **another Bimboolean** called Jane. Jane has lived in Bimboola all of her life.

Below are statements that will help you form an impression about Jane. Please take your time to carefully look through the statements. In the next section, you will be asked to describe Jane and your impression of her.

Jane works at an advertising company.

Jane was married but recently divorced.

Jane cannot afford a car.

On weekends, Jane visits her parents.

Jane has a dog.

Jane is not a morning person.

Jane cannot afford to go on holidays.

Jane likes to go to the gym after work.

Jane likes to eat cereal for breakfast.

Jane rides a bicycle to work.

Jane’s father is a farmer.

Jane has two children.

Jane's children go to a public school.

Jane has never been on an airplane.

Jane likes going to musicals.

Jane is from income group 3.

Jane enjoys public speaking.

Jane doesn’t like to eat broccoli.

Jane likes to eat rice.

Jane can speak two languages.

Jane's favourite class in high school was math.

**John_Rich_Statements**

In your daily life at Bimboola, you meet **another Bimboolean** called John. John has lived in Bimboola all of his life.

Below are statements that will help you form an impression about John. Please take your time to carefully look through the statements. In the next section, you will be asked to describe John and your impression of him.

John works at an advertising company.

John was married but recently divorced.

John owns an expensive sports car.

On weekends, John visits his parents.

John has a dog.

John is not a morning person.

John just booked a trip to go on a five-stared cruise.

John likes to go to the gym after work.

John likes to eat cereal for breakfast.

John rides a bicycle to work.

John’s father is a neurosurgeon.

John has two children.

John's children go to a prestigious private school.

John always travel first class on airplanes.

John likes going to musicals.

John is from income group 1.

John enjoys public speaking.

John doesn’t like to eat broccoli.

John likes to eat rice.

John can speak two languages.

John's favourite class in high school was math.

**John_Statements_Description task**

From what you know about John, how do you think he is as a person? **In 150-200 words, please describe your impression of him**. You can focus on the following:

1. What do you think is his usual daily routine? (e.g., his plan for a normal day)
2. Who do you think he usually interacts with? (e.g., his family, his colleagues)
3. What do you think would be his general mood and temperament? (e.g., is he an optimistic/a pessimistic person)

To what extent do you like or dislike John as a person?

- 1. (I dislike John very much)
  2. 3 4 5 6 7 (I like John

very much)

**John_Poor_Statements**

In your daily life at Bimboola, you meet **another Bimboolean** called John. John has lived in Bimboola all of his life.

Below are statements that will help you form an impression about John. Please take

your time to carefully look through the statements. In the next section, you will be asked to describe John and your impression of him.

John works at an advertising company.

John was married but recently divorced.

John cannot afford a car.

On weekends, John visits his parents.

John has a dog.

John is not a morning person.

John cannot afford to go on holidays.

John likes to go to the gym after work.

John likes to eat cereal for breakfast.

John rides a bicycle to work.

John’s father is a farmer.

John has two children.

John's children go to a public school.

John has never been on an airplane.

John likes going to musicals.

John is from income group 3.

John enjoys public speaking.

John doesn’t like to eat broccoli.

John likes to eat rice.

John can speak two languages.

John's favourite class in high school was math.

**Demographic questions**

We are now at the end of the study.

Lastly, based on your **real-life experience**, please take your time to truthfully answer the following demographic questions about yourself.

Please think of this ladder with 10 rungs as representing people with different levels of income, education, and occupational status in the US. People at the top of the ladder are those who earn the most amount of money, have the best education and job, whereas people at the bottom of the ladder are those who earn the least amount of money, have the worst education and job (or potentially no job).

Where would you place yourself on this ladder relative to others in the US? (Please respond based on the scale below)

| 1 (The | 2 | 3 | 4 | 5 | 6 | 7 | 8 | 9 | 10 (The |
| --- | --- | --- | --- | --- | --- | --- | --- | --- | --- |
| worst oﬀ) |  |  |  |  |  |  |  |  | best oﬀ) |

Please indicate the highest level of education you have completed (or are currently undertaking):

Please indicate your employment status:

Please indicate your personal annual income (before tax):

Please indicate your combined annual household income (before tax):

What is your ethnicity?

Please indicate your gender:

Male Female

Others. Please specify:

Prefer not to indicate

Please answer the following questions about your political orientation?

| 1 (Very | 2 | 3 | I am neutral | 5 | 6 | 7 (Very |
| --- | --- | --- | --- | --- | --- | --- |
| Liberal) |  |  |  |  |  | Conservative) |

1 (Left Wing) 2 3 I am neutral 5 6 7 (Right Wing)

Please specify your age:

Please specify your native language:

Powered by Qualtrics
