# Supplementary material for: The Language of Inequality: Evidence Economic Inequality Increases Wealth Category Salience
Source: Pers Soc Psychol Bull. 2021 Aug 5;48(8):1204–19. doi: 10.1177/01461672211036627 (PMC9245161; doi:10.1177/01461672211036627)
Supplement: sj-docx-4-psp-10.1177_01461672211036627 – Supplemental material for The Language of Inequality: Evidence Economic Inequality Increases Wealth Category Salience [file sj-docx-4-psp-10.1177_01461672211036627.docx]

# Participant sheet

## PARTICIPANT INFORMATION SHEET

The purpose of this study is to examine your thoughts and perceptions about wealth and social class in the United Kingdom. This study is being conducted by

### Participation and Withdrawal

Participation in this study is completely voluntary and you are free to withdraw at any time without any penalty. If you wish to withdraw, simply stop completing the survey. If you do choose to withdraw from the study, all materials that you have completed will be deleted and will not be included in the study.

### What is Involved?

This study has 3 parts. First, you will be asked to read about wealth and class in the UK and to then share your reflections in writing. Second, you will be asked to indicate what you think is important to know about other British people and then to describe another person's life. Third, you will be asked to describe your life in the UK. You will also be asked to provide some demographics. Participation in this study will take between 15 and 20 minutes. You will be reimbused GBP2.50 for participating.

*Please note that you are required to respond in writing to 3 open questions* - please do not participate in this study if you are not willing to provide high quality responses to these questions.

### Risks

Participation in this study should involve no physical or mental discomfort, and no risks beyond those of everyday living. If, however, you find any question or procedure to be uncomfortable or offensive, you are free to skip that aspect of the study.

### Confidentiality and security of data

All data collected in this study will be stored confidentially. Only members of the research team will have access to identified data. All data will be coded in a de-identified manner and subsequently analysed and reported in such a way that responses will not be able to be linked to any individual. The final data set will be fully anonymised by the removal of any potentially identifying information (including participant number). This fully anonymised data set will be archived on a publically available research transparency database such as the open science framework to enhance the openness and reproducibility of the research. Data will be collected and processed in line with EU GDPR guidelines.

### Ethics Clearance and Contacts

re participating, you can contact me at the email above, and I will send you an Abstract of the study and findings.

Thank you for your participation in this study.

**Clicking the button at the bottom of this screen confirms that you have read and understood the information provided above, and decided to participate as a research subject for this study.**

# Introduction

## WELCOME!

This study aims to explore your thoughts and perceptions about wealth and social class in the UK. We are interested in your actual experiences as well as your thoughts on how other British people live their lives.

There are 3 different parts to this study.


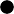

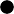
First, we will ask you to reflect on the UK’s economy and how it is ranked compared to other countries. After this, you will be asked to respond to some questions about this information. Second, we will ask you to indicate how important it is to know different kinds of information about other British people and then to write about the life of another British person.


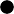
Third, we will ask you to write about your own life in the UK.

You will also be asked to provide your own demographics.

# GINI

## SECTION 1

**ECONOMIC INEQUALITY IN THE UK**

When we want to understand how a country is performing economically, there are several different economic indicators that we can use. In this section, we will focus on the **GINI coefficient**.

The GINI coefficient, named for its creator Corrado Gini, is used to measure a society’s economic inequality. The GINI coefficient takes values between 0 and 100.


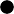
A value of 0 represents perfect equality, and would describe a country where everyone has the same income.


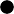
A value of 100 represents extreme inequality, and would describe a country where one person has all the income while everyone else has nothing.

Of course, no country will ever have a value of exactly 0 or 100; all countries will have values somewhere in between 0 and 100.

However, this does not mean that there aren't important differences between countries. Countries that have a relatively low GINI have an equal distribution of wealth, which means that the income of the richest and poorest people in a society does not differ by much. In contrast, countries that have a relatively high GINI have an unequal distribution of wealth, such that the income of the richest and poorest people in a society differs considerably.

When you are sure that you understand what the Gini measures, please click to advance to the next page.

# Unequal Condition

### THE UK's GINI COEFFICIENT

To understand how the UK is performing in terms of its economic inequality, it is helpful to compare its GINI coefficient with those of other countries. In the Figure below, we have used data from the most recent World Bank report on economic inequality to compare the performance of the UK with 5 other countries on the GINI coefficient.

The GINI coefficient is graphed on the vertical axis, and the higher the bar for a given country rises up this axis, the more unequal it is.


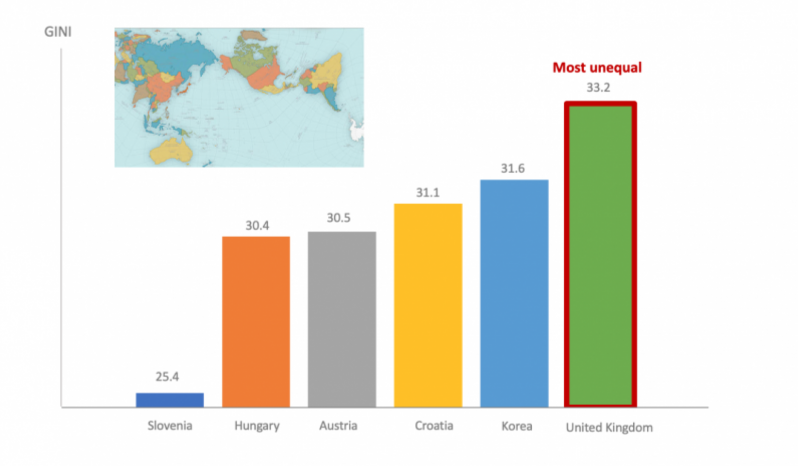


As you can see from this graph*,* Slovenia has the lowest GINI, with a value of 25.4, and is therefore the most EQUAL country. Indeed, Slovenia is one of the most equal countries in Europe. To give you a sense of what this means, in Slovenia, the top 20 percent of households only earn 3.6 times as much as the bottom 20 percent of households.

The UK is substantially more unequal than this. Indeed, as you can see from this graph, the UK has the highest GINI, with a value of 33.2, and is therefore the most UNEQUAL of these countries.

## REFLECTING ON INEQUALITY IN THE UK

Please take a moment to reflect on the information above before letting us know what this information means to you. How does the fact that the UK has a relatively high GINI, indicating higher levels of inequality, accord with your own experience of living in the UK?

Please provide your reflections in two or three sentences.

# Equal Condition

### THE UK's GINI COEFFICIENT

To understand how the UK is performing in terms of its economic inequality, it is helpful to compare its GINI coefficient with those of other countries. In the Figure below, we have used data from the most recent World Bank report on economic inequality to compare the performance of the UK with 5 other countries on the GINI coefficient.

The GINI coefficient is graphed on the vertical axis, and the higher the bar for a given country rises up this axis, the more unequal it is.


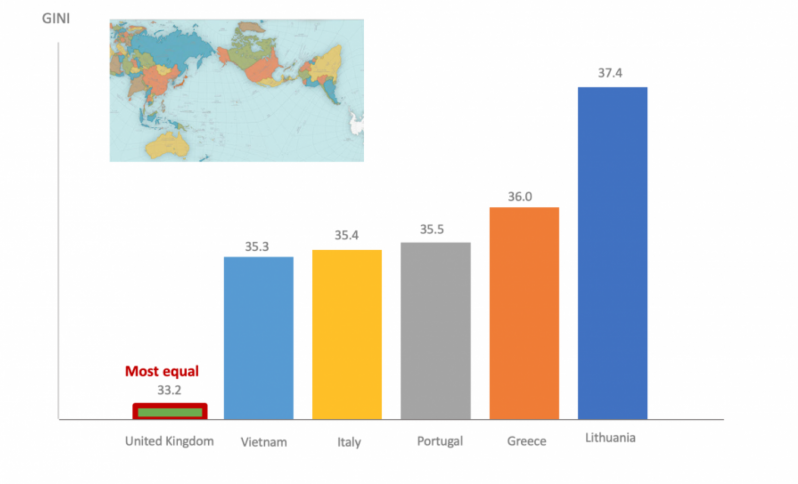


As you can see from this graph*,* Lithuania has the highest GINI, with a value of 37.4, and is therefore the most UNEQUAL country. Indeed, Lithuania is one of the most unequal countries in Europe. To give

you a sense of what this means, in Lithuania, the top 20 percent of households earn 7.5 times as much as the bottom 20 percent of households.

The UK is substantially more equal than this. Indeed, as you can see from the graph, the UK has the lowest GINI, with a value of 33.2, and is therefore the most EQUAL of these countries.

## REFLECTING ON EQUALITY IN THE UK

Please take a moment to reflect on the information above before letting us know what this information means to you. How does the fact that the UK has a relatively low GINI, indicating lower levels of inequality, accord with your own experience of living in the UK?

Please provide your reflections in two or three sentences.

# Manipulation Check, Wealth and Identity

## PERCEPTIONS OF INEQUALITY

Now, we'd like you to answer the following questions about how wealth is distributed in the UK.


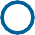

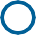

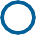

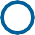

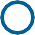

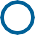
Overall, how small or large do you think that the wealth gap is between the poorest and wealthiest people in the UK?

| Very | Small | Somewhat | Neither | Somewhat | Large | Very |
| --- | --- | --- | --- | --- | --- | --- |
| Small |  | Small | Large nor | Large |  | Large |
|  |  |  | Small |  |  |  |


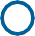


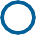

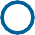

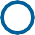

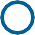

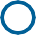

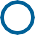
In your view, to what extent has the wealth gap between the poorest and richest people in the UK decreased or increased in recent years?

| Decreased | Decreased | Decreased | Neither | Increased | Increased | Increased |
| --- | --- | --- | --- | --- | --- | --- |
| a lot |  | a little | Increased | a Little |  | a lot |
|  |  |  | nor |  |  |  |
|  |  |  | Decreased |  |  |  |


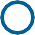


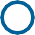

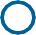

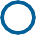

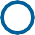

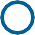

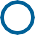
How much do you agree that the gap between rich and poor people in the UK is so large that it is as if they live in different worlds?

| Strongly | Disagree | Slightly | Neither | Slightly | Agree | Strongly |
| --- | --- | --- | --- | --- | --- | --- |
| Disagree |  | Disagree | Agree nor | Agree |  | Agree |
|  |  |  | Disagree |  |  |  |


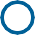


How fair do you think is the wealth distribution between the poorest and wealthiest people in the UK?

Very Unfair Unfair Slightly


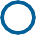

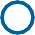
Unfair

Neither Fair nor Unfair

Slightly Fair Fair Very Fair


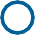

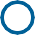


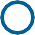

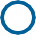

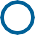

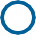
In your view, to what extent is the gap between the poorest and wealthiest people in the UK justifiable?

Totally Unjustifiable


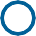


Unjustifiable Slightly


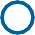
Unjustifiable


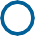


Neither Justifiable nor Unjustifiable

Slightly Justifiable


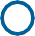


Justifiable Totally Justifiable


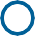


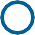


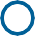

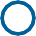

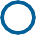

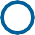

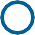

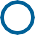
How much do you agree that the gap in wealth between rich and poor people in the UK is legitimate?

| Strongly | Disagree | Slightly | Neither | Slightly | Agree | Strongly |
| --- | --- | --- | --- | --- | --- | --- |
| Disagree |  | Disagree | Agree nor | Agree |  | Agree |
|  |  |  | Disagree |  |  |  |


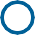


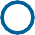

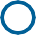

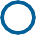

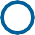

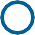

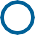
How much do you agree that the wealth gap in the UK is the result of people getting what they deserve?

| Strongly | Disagree | Slightly | Neither | Slightly | Agree | Strongly |
| --- | --- | --- | --- | --- | --- | --- |
| Disagree |  | Disagree | Agree nor | Agree |  | Agree |
|  |  |  | Disagree |  |  |  |


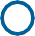


# Intro to Bimboolean Chris M

## SECTION 2

**LIVING IN THE UK**

Now, we'd like you to imagine that in the course of your day-to-day life you meet another British person called Chris.


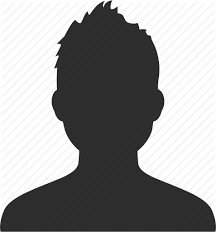


### Imagine that you want to get an idea of what Chris is like as a person.

There are, of course, many pieces of information that you could use to get a better idea of what Chris is like. We have provided a list of potential pieces of information below. You may feel that some of these pieces of information are *very important* for getting to know what Chris is like, and some other pieces of information are *not at all important* in forming a judgement of Chris.

For each of the pieces of information below, please indicate how important you think it is for knowing what Chris is like.

It is very important to me that I know if Chris is..

Strongly

No No Neutral Yes

Strongly Yes

Righteous
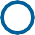

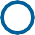

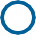

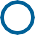

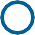
 Trustworthy
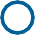

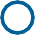

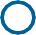

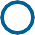

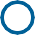
 Respectful
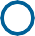

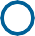

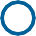

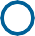

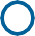
 Sincere
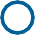

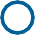

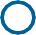

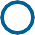

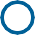
 Honest
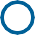

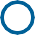

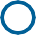

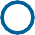

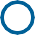


It is very important to me that I know if Chris is..

Strongly

No No Neutral Yes

Strongly Yes

Helpful
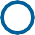

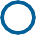

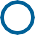

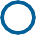

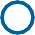
 Friendly
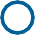

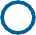

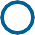

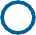

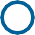
 Kind
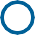

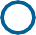

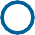

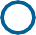

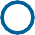
 Likeable
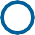

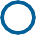

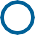
 Warm

It is very important to me that I know if Chris is..

Strongly

No No Neutral Yes

Strongly Yes

Capable Intelligent Skilful Competent Eﬃcient

It is very important to me that I know of Chris's..

Strongly

No No Neutral Yes

Strongly Yes

Hobby Salary Education Ethnicity Occupation Political View Age Social Class Religion

It is very important to me that I know if Chris is..

|  | Strongly No | No | Neutral | Yes | Strongly Yes |
| --- | --- | --- | --- | --- | --- |
| A caring person who cherishes and protects others |  |  |  |  |  |
| A fair person who will never cheat or break the rules |  |  |  |  |  |
| A loyal person who will stand with other people |  |  |  |  |  |
| A respectful person who will uphold traditions and obey authority |  |  |  |  |  |
| A person who is physically and spiritually clear and pure |  |  |  |  |  |
| A ... This is a control question, please select "Strongly No" |  |  |  |  |  |

# John_Rich_Statements

### LIVING IN THE UK, continued.

Now, imagine that in the course of your day-to-day life in the UK, you meet another British individual called John.

Your will find some statements that will help you to form an impression of John below. Please take your time to carefully look through the statements. In the next section, you will be asked to describe John and your impressions of him.

John works at an advertising company. John was married but recently divorced. John owns an expensive sports car.

On weekends, John visits his parents. John has a dog.

John is not a morning person.

John just booked a trip to go on a five-stared cruise. John likes to go to the gym after work.

John likes to eat cereal for breakfast. John rides a bicycle to work.

John’s father is a neurosurgeon. John has two children.

John's children go to a prestigious private school. John always travel first class on airplanes.

John likes going to musicals. John is wealthy.

John enjoys public speaking. John doesn’t like to eat broccoli. John likes to eat rice.

John can speak two languages.

John's favourite class in high school was math.

# John_Statements_Description task

### What do you think that John is like as a person?

In 150-200 words, please describe your impression of John and his daily life in the UK. In your description, you may want to describe the following:

His daily routine (e.g., his plan for a normal day)

The people he usually interacts with (e.g., his family, his colleagues)

His general mood and temperament (e.g., is he an optimistic/a pessimistic person)

# John_Poor_Statements

### LIVING IN THE UK, continued.

Now, imagine that in the course of your day-to-day life in the UK, you meet another British individual called John.

Your will find some statements that will help you to form an impression of John below. Please take your time to carefully look through the statements. In the next section, you will be asked to describe John and your impressions of him.

John works at an advertising company. John was married but recently divorced. John cannot afford a car.

On weekends, John visits his parents. John has a dog.

John is not a morning person.

John cannot afford to go on holidays. John likes to go to the gym after work. John likes to eat cereal for breakfast. John rides a bicycle to work.

John’s father is a farmer. John has two children.

John's children go to a public school. John has never been on an airplane. John likes going to musicals.

John is poor.

John enjoys public speaking. John doesn’t like to eat broccoli. John likes to eat rice.

John can speak two languages.

John's favourite class in high school was math.

# Intro Description Task

## SECTION 3:

**YOUR LIFE IN THE UK**

In this section, we're interested in learning about how you find living in the UK. For this, we would like you to reflect on your daily life and then write about it in the box below. Please aim for around

150-200 words.

In your description, you may wish to write about the following topics:

Your ***daily activities*** (i.e., outline your plans for the day).

Your main ***social interactions*** (i.e., the individuals you would meet, their relationships to you, how you would interact with them and the tone of the interaction).

Your ***thoughts and feelings*** throughout your day (i.e., how you would feel waking up, going about your daily activities and meeting the individuals you have described).

# Demographic questions

## DEMOGRAPHICS

In this final section, please answer the following demographic questions about yourself.

### Social Ladder

Please think of this ladder with 10 rungs as representing people with different levels of income, education, and occupational status in the UK. People at the top of the ladder are those who earn the most amount of money, have the best education and job, whereas people at the bottom of the ladder are those who earn the least amount of money, have the worst education and job (or potentially no job).

Where would you place yourself on this ladder relative to others in the UK? (Please respond based on the scale below)

10 (The BEST oﬀ)

9

8

7

6

5

4

3

2

1 (The WORST oﬀ)

Please indicate the highest level of education you have completed (or are currently undertaking):

Please indicate your employment status:

Please indicate your personal annual income (before tax):

What is your ethnicity?

What is your gender?

Male Female

Non-binary / third gender

Other (prefer to self-define):

What is your political orientation from left wing to right wing?

| 1 | 2 | 3 | 4 | 5 | 6 | 7 |
| --- | --- | --- | --- | --- | --- | --- |
| (Left Wing) |  |  | (I am neutral) |  |  | (Right Wing) |

What is your political orientation from liberal to conservative?

1

(Very Liberal)

2 3 4

(I am neutral)

5 6 7

(Very Conservative)

What is your age (in years):

What is your native language?

At the beginning of this study, we directly contrasted the economic inequality in the UK with the economic inequality in another European country. Was this country "Scotland"?

Yes No

What is your Prolific ID?

${e://Field/PROLIFIC_PID}

Powered by Qualtrics
