# Supplementary material for: The Language of Inequality: Evidence Economic Inequality Increases Wealth Category Salience
Source: Pers Soc Psychol Bull. 2021 Aug 5;48(8):1204–19. doi: 10.1177/01461672211036627 (PMC9245161; doi:10.1177/01461672211036627)
Supplement: sj-docx-5-psp-10.1177_01461672211036627 – Supplemental material for The Language of Inequality: Evidence Economic Inequality Increases Wealth Category Salience [file sj-docx-5-psp-10.1177_01461672211036627.docx]

## Participant info

Welcome to this study!

# Participant Information Sheet

The purpose of this study is to examine your experience living in Australia. This study is being conducted by

# Participation and Withdrawal

Participation in this study is completely voluntary and you are free to withdraw at any time without any penalty. If you wish to withdraw, simply stop completing the survey. If you do choose to withdraw from the study, all materials that you have completed will be deleted and will not be included in the study.

# What is Involved?

Participants will be asked to respond to several questions about what life in Australia is like for themselves and other people. Participation in this study will take 10-20 minutes.

# Risks

Participation in this study should involve no physical or mental discomfort, and no risks beyond those of everyday living. If, however, you find any question or procedure to be uncomfortable or offensive, you are free to omit from answering or participating in that aspect of the study.

# Confidentiality and Security of Data

All data collected in this study will be stored confidentially. Only members of the research team will have access to the data. All data will be coded in a de-identified manner and subsequently analysed and reported in such a way that responses will not be able to be linked to any individual. The data you provide will only be used for the research purpose of this study.

# Ethics Clearance and Contacts

This study has been cleared in accordance with the ethical review processes of the University of

If you would like to learn the outcome of the study in which you are participating, you can contact me using the email above and I will send you an Abstract of the study and findings.

Thank you for your participation in this study.

## Age, Gender and Location

Before we begin, could you please specify the following information:

What is your age?


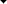


What is your gender?


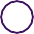
 Male
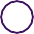
 Female


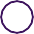


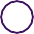
 Prefer not to say

Others, please specify

Which Australian state are you residing in?


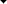


## Begin

**Let's Begin!**

This study aims to explore your **thoughts and perceptions about wealth and social class in Australia**. We are interested in your actual experiences as well as your thoughts on how other Australians live their lives.

There will be 3 different parts to this study. Firstly, we will ask you some general questions about yourself and how you perceive Australia as a society. Next, you will be asked to reflect on your own life and write about your daily experiences. In the last section, you will be asked to think about other Australians and how their experiences might be similar or different to your own. Please take your time to answer each part truthfully.

## Section B. Perceived Inequality in Society

To start off, we would like you to answer the following questions about **how wealth is distributed in Australia.**

Overall, how small or large is the wealth gap between the poorest and the wealthiest people in Australia?

Very Small Small Somewhat


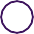

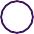

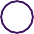

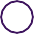
Small

Neither Large nor Small

Somewhat Large

Large Very Large


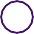

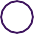


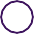


Overall, would you like the current wealth gap between the poorest and the wealthiest to be smaller or larger?

Much Smaller

Smaller Somewhat


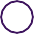
Smaller

Remain the same

Somewhat Larger

Larger Much Larger


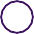

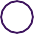

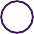

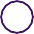


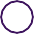

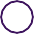
In your view, to what extent has the wealth gap between the poorest and the wealthiest people in Australia decreased or increased over the last decades?

Decreased a lot

Decreased Decreased a


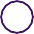
little

Remained the same

Increased a little

Increased Increased a


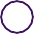
lot


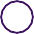

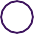

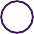

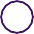

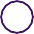


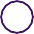

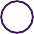

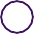
How much do you agree that the gap between rich and poor Australians is so large that it is as if they live in different worlds?

| Strongly | Disgree | Somewhat | Neutral | Somewhat | Agree | Strongly |
| --- | --- | --- | --- | --- | --- | --- |
| Disagree |  | Disgree |  | Agree |  | Agree |


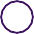

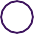

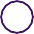

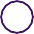


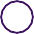

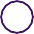

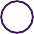

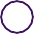

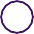
How fair is the wealth distribution between the poorest and the wealthiest people in Australia?

| Very Unfair | Unfair | Somewhat | Neutral | Somewhat | Fair | Very Fair |
| --- | --- | --- | --- | --- | --- | --- |
|  |  | Unfair |  | Fair |  |  |


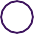

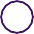


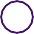
In your view, to what extent is the gap between the poorest and the wealthiest people in our country justifiable?

Totally Unjustifiable

Unjustifiable Somewhat


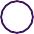
Unjustifiable

Neutral Somewhat


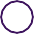
Justifiable

Justifiable Totally Justifiable


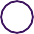

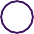

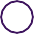

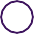


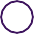

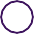

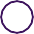

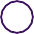

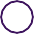

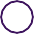
How much do you agree that the gap in wealth between rich and poor Australians is legitimate?

| Strongly | Disagree | Somewhat | Neither | Somewhat | Agree | Strongly |
| --- | --- | --- | --- | --- | --- | --- |
| Disagree |  | Disagree | Agree nor | Agree |  | Agree |
|  |  |  | Disagree |  |  |  |


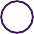


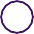

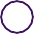

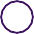

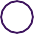

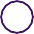

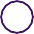
How much do you agree that the wealth gap in Australia is the result of people getting what they deserve?

| Strongly | Disagree | Somewhat | Neither | Somewhat | Agree | Strongly |
| --- | --- | --- | --- | --- | --- | --- |
| Disagree |  | Disagree | Agree nor | Agree |  | Agree |
|  |  |  | Disagree |  |  |  |


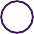


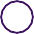

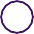

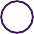

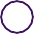
Relative to others in Australia, how would you classify your own wealth?

| Very poor | Poor | Somewhat | Netural | Somewhat | Wealthy | Very |
| --- | --- | --- | --- | --- | --- | --- |
|  |  | Poor |  | Wealthy |  | Wealthy |


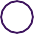

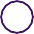

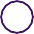


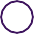

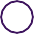

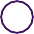
In 10 years' time, do you think that **you** will be much poorer, about the same, same as now, or much wealthier?

| Much Poorer | Poorer | Somewhat | About the | Somewhat | Wealthier | Much |
| --- | --- | --- | --- | --- | --- | --- |
|  |  | Poorer | Same | Wealthier |  | Wealthier |


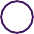

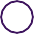

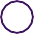

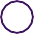


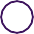

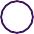

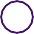
In 10 years’ time, do you expect **Australia** to be much poorer, about the same, same as now or much wealthier?

| Much Poorer | Poorer | Somewhat | About the | Somewhat | Wealthier | Much |
| --- | --- | --- | --- | --- | --- | --- |
|  |  | Poorer | Same | Wealthier |  | Wealthier |


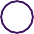

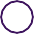

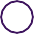

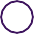


Now, we would like you to think of 100 citizens of Australia. If Australia constitutes of these 100 citizens, **in your view, how many of these 100 people would be classified as “very poor”, “poor”, “average in wealth”, “wealthy”, “very wealthy”?**

**Please provide an estimate of the number of people in each box. Please make sure that the total estimate adds up to 100 people.**

Very poor

0

Poor

0

Average in wealth

0

Wealthy

0

Very wealthy

0

Total

0

## Demographic questions

Next, we would like you think about **your own life in Australia.** Please take your time to truthfully answer the following demographic questions about yourself.

Please indicate your employment status:


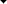


Please indicate your personal annual income (before tax):


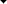


Please indicate your combined annual household income (before tax):


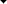


Please indicate the highest level of education you have completed (or are currently undertaking):


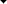


Please think of this ladder with 10 rungs as representing people with different levels of income, education, and occupational status in Australia. People at the top of the ladder are those who earn the most amount of money, have the best education and job, whereas people at the bottom of the ladder are those who earn the least amount of money, have the worst education and job (or potentially no job).


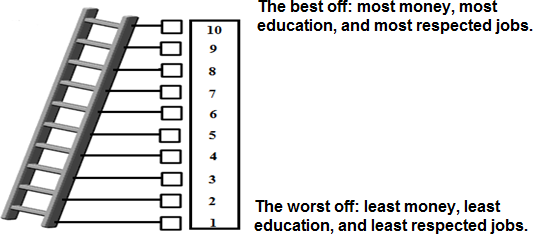
Where would you place yourself on this ladder relative to others in society? (Please respond based on the scale below)

1 (The worst off)

2 3 4 5 6 7 8 9 10 (The


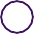

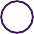

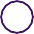

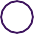

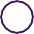

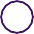

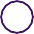

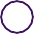

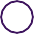
best off)


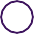


## Intro Description Task

In the previous questions, we have asked you general questions about wealth and social class in Australia.

Now, we would like you to specifically think about your own daily life and how it may be the same or different to other Australians. **In 150-200 words, describe what life is like for you.**

Generally, describe how your normal day would unfold. You can talk about: 1.) Your ***daily activities*** (i.e., outline your priorities and plans for the day).

2.) Your main ***social interactions*** (i.e., the social groups or individuals you meet, their relationships to you, how you interact with them and the tone of the interaction).

3.) Your ***thoughts and feelings*** throughout your day in Australia (i.e., how you would feel waking up, going about your daily activities and meeting the individuals you have described). You can also talk about your goals and motivations.

## Introduction to other Australian task

Thank you for providing the description of your own life. The following sections will ask you to think about other Australians and their life.

**Intro to Christine**

# Imagine that one day you meet another Australian citizen named Christine.


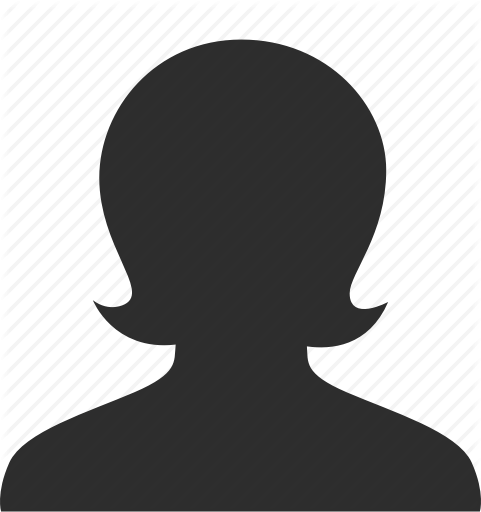


## Christine_Getting to know task

Now imagine that you want to get an idea of what Christine is like as a person.

There are, of course, many pieces of information that you could use to get a better idea of what Christine is like. We have provided a list of potential pieces of information below. You may feel that some of these pieces of information are *very important* for

getting to know what Christine is like, and some other pieces of information are *not at all important* in forming a judgement of Christine.

For each of the information, please indicate how important you think it is for knowing what Christine is like.

## "It is very important to me that I know if Christine is..."

Strong No No Neutral Yes Strong Yes Righteous
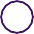
 Sincere Respectful Honest Trustworthy

## "It is very important to me that I know if Christine is..."

Strong No No Neutral Yes Strong Yes Likeable Helpful Friendly Kind

Warm

## "It is very important to me that I know if Christine is..."

Strong No No Neutral Yes Strong Yes Skillful Capable Intelligent Efficient Competent

## "It is very important to me that I know Christine's.."

Strong No No Neutral Yes Strong Yes Occupation Hobby

Age Ethnicity Education

Socioeconomic Status

Religion Political view Salary

## "It is very important to me that I know that Christine is..."

Strong No No Neutral Yes Strong Yes

A caring person,

who cherishes and protects others

A fair person, who

will never cheat or break the rules

A loyal Australian,

who will stand with other Australians

A respectful person, who will uphold traditions and obey authority

A person who is physically and spiritually clear and pure

A ... This is a control question, please select "Strongly No"

## John_Rich_Statements

In your daily life in Australia, you meet **another Australian** called John.

Below are statements that will help you form an impression about John. Please take your time to carefully look through the statements. In the next section, you will be asked to describe John and your impression of him.

John works at an advertising company.

John was married but recently divorced.

John owns an expensive sports car.

On weekends, John visits his parents.

John has a dog.

John is not a morning person.

John just booked a trip to go on a five-stared cruise.

John likes to go to the gym after work.

John likes to eat cereal for breakfast.

John rides a bicycle to work.

John’s father is a neurosurgeon.

John has two children.

John's children go to a prestigious private school.

John always travel first class on airplanes.

John likes going to musicals.

John is wealthy.

John enjoys public speaking.

John doesn’t like to eat broccoli.

John likes to eat rice.

John can speak two languages.

John's favourite class in high school was math.

## John_Statements_Description task

From what you know about John, how do you think he is as a person? **In 150-200 words, please describe your impression of him**. You can focus on the following:

1. What do you think is his usual daily routine? (e.g., his plan for a normal day)
2. Who do you think he usually interacts with? (e.g., his family, his colleagues)
3. What do you think would be his general mood and temperament? (e.g., is he an optimistic/a pessimistic person)

To what extent do you like or dislike John as a person?

1 (I dislike John very much)

2 3 4 5 6 7 (I like John

very much)

## John_Poor_Statements

In your daily life in Australia, you meet **another Australian** called John.

Below are statements that will help you form an impression about John. Please take your time to carefully look through the statements. In the next section, you will be asked to describe John and your impression of him.

John works at an advertising company.

John was married but recently divorced.

John cannot afford a car.

On weekends, John visits his parents.

John has a dog.

John is not a morning person.

John cannot afford to go on holidays.

John likes to go to the gym after work.

John likes to eat cereal for breakfast.

John rides a bicycle to work.

John’s father is a farmer.

John has two children.

John's children go to a public school.

John has never been on an airplane.

John likes going to musicals.

John is poor.

John enjoys public speaking.

John doesn’t like to eat broccoli.

John likes to eat rice.

John can speak two languages.

John's favourite class in high school was math.

## Immigrants

In this section of the survey we are interested in your perception of immigration into Australia. Please respond to each question truthfully.

Immigrants take resources and employment opportunities away from Australia.

| Strongly | Disagree | Somewhat | Neither | Somewhat | Agree | Strongly |
| --- | --- | --- | --- | --- | --- | --- |
| Disagree |  | Disagree | Agree nor | Agree |  | Agree |
|  |  |  | Disagree |  |  |  |

In school where there are too many children of immigrants, the quality of education will suffer.

| Strongly | Disagree | Somewhat | Neither | Somewhat | Agree | Strongly |
| --- | --- | --- | --- | --- | --- | --- |
| Disagree |  | Disagree | Agree nor | Agree |  | Agree |
|  |  |  | Disagree |  |  |  |

Immigrants abuse the system of social benefits.

| Strongly | Disagree | Somewhat | Neither | Somewhat | Agree | Strongly |
| --- | --- | --- | --- | --- | --- | --- |
| Disagree |  | Disagree | Agree nor | Agree |  | Agree |
|  |  |  | Disagree |  |  |  |

Australian norms and values are being threatened by the presence of immigrants.

| Strongly | Disagree | Somewhat | Neither | Somewhat | Agree | Strongly |
| --- | --- | --- | --- | --- | --- | --- |
| Disagree |  | Disagree | Agree nor | Agree |  | Agree |
|  |  |  | Disagree |  |  |  |

The cultural practices of immigrants threaten the Australian way of life.

| Strongly | Disagree | Somewhat | Neither | Somewhat | Agree | Strongly |
| --- | --- | --- | --- | --- | --- | --- |
| Disagree |  | Disagree | Agree nor | Agree |  | Agree |
|  |  |  | Disagree |  |  |  |

Immigrants are a threat to Australian identity.

| Strongly | Disagree | Somewhat | Neither | Somewhat | Agree | Strongly |
| --- | --- | --- | --- | --- | --- | --- |
| Disagree |  | Disagree | Agree nor | Agree |  | Agree |
|  |  |  | Disagree |  |  |  |

## More demographic questions

We have almost reached the end of the survey. Please answer the following demographic questions truthfully.

Please answer the following questions about your political orientation:

1

(Very Liberal)

2 3 4

I am neutral

5 6 7

(Very Conservative)

| 1 | 2 | 3 | 4 | 5 | 6 | 7 |
| --- | --- | --- | --- | --- | --- | --- |
| (Left Wing) |  |  | I am neutral |  |  | (Right Wing) |

What is your native language ?

Powered by Qualtrics
