# Supplementary material for: The Language of Inequality: Evidence Economic Inequality Increases Wealth Category Salience
Source: Pers Soc Psychol Bull. 2021 Aug 5;48(8):1204–19. doi: 10.1177/01461672211036627 (PMC9245161; doi:10.1177/01461672211036627)
Supplement: sj-docx-6-psp-10.1177_01461672211036627 – Supplemental material for The Language of Inequality: Evidence Economic Inequality Increases Wealth Category Salience [file sj-docx-6-psp-10.1177_01461672211036627.docx]

**The Language of Inequality: Supplementary Analysis**

This document provides the following information and analysis:

- Study 1
  - Original 74 Wealth Category Word Dictionary
  - Analysis controlling for poverty levels
- Study 2
  - Analysis controlling for poverty levels
  - Analysis including additional economic indicator as controls
- Study 3a
  - Additional manipulation check
  - Exploratory attribute analyses
  - Analysis of inequality as a social problem
  - LIWC wealth category code analysis
- Study 3b
  - Additional manipulation check
  - Exploratory attribute analyses
  - Analysis of inequality as a social problem
  - LIWC wealth category code analysis
- A note on Study 3a & 3b exclusion rates
- Study 5 survey of subjective inequality and wealth category references in Australia.
- References

**Study 1**

**Original 74 Wealth-Category Word Dictionary:**

**Rich words**: independent, easy, rich, grand, luxury, fat, swimming, wealthy, loaded, fancy, ample, stylish, privileged, affluent, lavish, prosperous, posh, flush, classy, new money, well-off, plush, in the money, well-heeled, gilded, opulent, uptown, embellished, well-to-do, high-class, fortuitous, resplendent, snazzy, old money, well-endowed, filthy rich, spiffy, nouveau riche, rolling in it, well provided for, made of money, pecunious.

**Poor words**: poor, impoverished, destitute, indigent, meagre, penniless, poverty-stricken, underprivileged, bankrupt, insolvent, scanty, bad off, beggared, beggarly, dirt poor, empty-handed, behind the eight ball, broke, flat broke, fortuneless, impecunious, moneyless, necessitous, pauperized, penurious, stone broke, cash-strapped, truly needy, unprosperous, insufficient, diminutive, exiguous.

**Controlling for Poverty Levels**

Table S1. Unstandardised coefficients of the association between economic indicators and percentage of wealth category references in UK and US books, controlling for national poverty.

|  |  | UK | |  |  | US | |  |
| --- | --- | --- | --- | --- | --- | --- | --- | --- |
|  | Step 1 | | Step 2 | | Step 1 | | Step 2 | |
| Year | -.002***  (.001) | | -.003***  (.001) | | -.008***  (.001) | | -.007***  (.002) | |
| GDP | .035***  (.010) | | .033**  (.010) | | .164***  (.032) | | .099*  (.039) | |
| Inequality | .015**  (.005) | | .021**  (.007) | | -.020*  (.009) | | .008  (.013) | |
| Poverty |  | | -.005  (.004) | |  | | -.016**  (.005) | |
| Constant | .168  (.164) | | .178  (.164) | | .376  (.163) | | .340*  (.163) | |
| Observations | 1,610 | | 1,610 | | 1,750 | | 1,750 | |

*Notes.* Poverty data available from 1959 (US) and 1961 (UK); US data is sourced from the US Census Bureau and measures the proportion of households earning less than a threshold value; UK data is sourced from UK Institute for Fiscal Studies and measures the proportion of the population earning less than 50% of the median income.

**Study 2**

**Controlling for Poverty Levels**

Table S2. Unstandardised regression coefficients of the economic indicators on prevalence of wealth category words controlling for national poverty.

|  | Step 1 | Step 2 | Step 3 |
| --- | --- | --- | --- |
| Year | -.000  (.000) | .001**  (.000) | .001**  (.000) |
| GDP |  | -.045***  (.004) | -.043***  (.004) |
| GDP (CRE) |  | .055  (.050) | .056  (.051) |
| Gini | .038**  (.012) | .099***  (.013) | .087***  (.015) |
| Gini (CRE) | -.003  (.030) | -.067*  (.031) | -.064  (.041) |
| Poverty |  |  | .020  (.011) |
| Poverty (CRE) |  |  | -.009  (.033) |
| Constant | 0.332***  (.040) | 0.285***  (.041) | 0.284***  (.043) |
| Obs | 5,810 | 5,810 | 5,810 |

*Notes.* * *p*<.050, ** *p*<.010, *** *p*<.001. All predictors are standardised. CRE = clustered random error term. Countries’ poverty data are for the most part obtained from national bodies (US Census Bureau, UK Institute for Fiscal Studies, Statistics Canada, Ireland Central Statistics Office, World Bank, Hong Kong Census and Statistics Department, ACOSS, NZ Ministry of Social Development, Statistics South Africa). We were able to source national poverty rate data for some years in all countries except Singapore. We obtained data for 186 country-years (an average of 16.91 years per country, ranging from 4 to 42 years).

**Comparing Alternative Economic Indicators**

Table S3. Unstandardised regression coefficients of the economic indicators on prevalence of wealth category words among countries with disposable or gross Gini coefficients.

|  | Step 1 | Step 2 | Step 3 | Step 1 | Step 2 | Step 3 |
| --- | --- | --- | --- | --- | --- | --- |
| Year | .000  (.000) | .004***  (.001) | .003***  (.001) | .000  (.000) | .002***  (.001) | -.002**  (.001) |
| GDP |  | -.031***  (.004) | -.040***  (.005) |  | .136***  (.024) | .126***  (.024) |
| GDP (CRE) |  | .044  (.067) | .053  (.067) |  | -.088  (.391) | .331  (.874) |
| GDP growth |  | .081***  (.014) | .070***  (.014) |  | .080***  (.011) | .075***  (.011) |
| GDP growth (CRE) |  | .112  (.393) | .131  (.395) |  | .025  (.369) | .562  (1.075) |
| GDP per capita |  | -.021*  (.010) | -.026**  (.010) |  | -.046***  (.006) | -.042***  (.006) |
| GDP per capita (CRE) |  | .010  (.107) | .021  (.113) |  | .022  (.356) | -.302  (.719) |
| GDP per capita growth |  | -.071***  (.013) | -.063***  (.013) |  | -.074***  (.010) | -.070***  (.010) |
| GDP per capita growth (CRE) |  | -.181  (.537) | -.193  (.542) |  | .098  (.415) | -.220  (.727) |
| Gini (gross) | .019  (.012) |  | .062***  (.013) |  |  |  |
| Gini (disposable) |  |  |  | .037***  (.006) |  | .026***  (.006) |
| Gini (CRE) | .005  (.040) |  | -.056  (.048) | -.013  (.047) |  | -.121  (.181) |
| Constant | 0.343***  (.055) | 0.252***  (.054) | 0.256***  (.058) | 0.307***  (.049) | 0.304  (.222) | 0.601  (.567) |
| Obs | 5,530 | 5,530 | 5,530 | 4,970 | 4,970 | 4,970 |

*Notes.* * *p*<.050, ** *p*<.010, *** *p*<.001. CRE = clustered random error term. Countries included in the analysis with gross Gini are Canada, Hong Kong, India, Malaysia, Philippines, Singapore, South African, the UK and the US; countries included in the analysis with disposable Gini are Australia, Canada, Ireland, New Zealand, Singapore and the UK.

**Study 3**

**Additional Manipulation Check**

We also ran a 2 (inequality condition: high or low, between) X 3 (target group: 1, 2 or 3, within) mixed ANOVA of participants’ ratings of the wealth of each of the income groups. This revealed main effects of condition, F(1,224)=5.11, p=.025, η2=.02, and income group, F(2,448)=461.40, p<.001, η2=.65, as well as their two-way interaction, F(2,448)=24.70, p<.001, η2=.03. In line with expectations, simple effects analysis showed that relative to participants in the low inequality condition, those in the high inequality condition perceived group 1 to be significantly more wealthy (low M=7.27, SD=1.48; high M=8.16, SD=1.83), F(1,224)=16.30, p<.001, η2=.07, and group 3 to be significantly less wealthy (low M=3.37, SD=1.73; high M=1.94, SD=2.23), F(1,224)=28.53, p<.001, η2=.11. In contrast, perceptions of group 2’s wealth did not vary significantly across conditions (low M=5.25, SD=0.70; high M=5.24, SD=0.75), F(1,224)=0.01, p=.931, η2=.00.3 In sum, then, these analyses show that we were successful in manipulating participants’ perceptions of inequality.

Of note, the finding that perceptions of group 2’s wealth did not vary significantly across conditions is inconsistent with the finding of Sanchez et al. (2019) who found that participants with average wealth perceived they were less wealthy in the high compared to the low inequality condition.

Studies 3 and 4 also measured participants’ identification with Bimboola and their own group. Identification was significantly higher in the equal condition.

**Attribute Importance Ratings**

For exploratory purposes, we followed Brambilla, Sacchi, Rusconi, Cherubini and Yzerbyt (2011) by asking how important it was to know if the individual was moral (α=.80), warm (α=.87) and competent (α=.86), with each dimension represented by 5 items: “sincere”, “honest”, “righteous”, “trustworthy” and “respectful”; “kind”, “friendly”, “warm”, “likeable” and “helpful”; “intelligent”, “competent”, “efficient”, “skillful”, and “capable”. Finally, participants were also asked to rate the importance of knowing about the extent to which an individual satisfied each of Haidt et al.’s (2009) moral foundations: “A caring person, who cherishes and protects others”, “A fair person, who will never cheat or break the rules”, “A loyal Bimboolean, who will stand with other Bimbooleans”, “A respectful person, who will uphold traditions and obey authority”, “A person who is physically and spiritually clear and pure”. All items were accompanied by identical 5-point Likert scales (1=strongly no, 5=strongly yes). Evidence that different moral foundations are differentially linked with situational individualism and collectivism (e.g., Yilmaz, Harma, Bahcekapili & Cesur, 2016) raises the possibility that they may be affected by income inequality too.

We conducted a series of independent samples t-tests to compare participants’ ratings of the importance of knowing information that was more (versus less) strongly related to wealth as a function of experimental condition. Experimental condition did not have any significant impact on concern about a stranger’s morality, warmth or competence, all *t*(224)$\leq$1.74, *p*$\geq.$083, or with one exception, their alignment with the moral foundations, all *t*(224)$\leq$1.73, *p*$\geq.$085. The exception occurred for ratings of the importance of being a loyal Bimboolean, as participants in the low inequality condition said that this information was more important than those in the high inequality condition (low *M*=3.66, *SD*= 0.91; high *M*=3.19, *SD*= 1.01), *t*(224)=3.70, *p*<000.

**Inequality as a Social Problem**

Table S4. Logistic regression coefficients of experimental condition and negative and positive affective terms on prevalence of wealth category words, *Own* Life.

|  | Group 1 | | Group 2 | | Group 3 | |
| --- | --- | --- | --- | --- | --- | --- |
|  | Step 1 | Step 2 | Step 1 | Step 2 | Step 1 | Step 2 |
| Inequality Condition | 0 .508  (.309) | 0.234  (.335) | 0.779**  (.273) | 0.546  (.289) | 1.059**  (.312) | 0.899*  (.348) |
| Positive words |  | -0.132*  (.065) |  | -0.165**  (.055) |  | -0.087  (.064) |
| Negative words |  | 0 .471***  (.127) |  | 0.289*  (.122) |  | 0.714***  (.143) |
| Constant | -1.319***  (.235) | -1.064*  (.430) | -0.625**  (.201) | 0.017  (.368) | -1.493***  (.247) | -1.750***  (.458) |

*Notes.* * *p*<.050, ** *p*<.010, *** *p*<.001. Inequality condition is a dummy variable with a value of 1 in the inequality condition and 0 otherwise. Positive / negative words is the percentage of words falling into default positive / negative affect dictionaries of the software LIWC (Pennebaker et al., 2007).

Table S5. Logistic regression coefficients of experimental condition and negative and positive affective terms on prevalence of wealth category words, *Citizen* Life.

|  | Group 1 | | Group 2 | | Group 3 | |
| --- | --- | --- | --- | --- | --- | --- |
|  | Step 1 | Step 2 | Step 1 | Step 2 | Step 1 | Step 2 |
| Inequality Condition | 0 .825*  (.352) | 0.749*  (.357) | 1.235**  (.461) | 1.166*  (.467) | 1.010**  (.309) | 0.975**  (.316) |
| Target Wealthy | 3.017  (.423) | 3.113***  (.435) | 0.973*  (.433) | 1.059*  (.442) | -1.390***  (.313) | -1.421***  (.322) |
| Positive words |  | -0.067  (.060) |  | -0.135  (.080) |  | -0.068  (.053) |
| Negative words |  | 0.154  (.125) |  | 0.116  (.130) |  | 0.216*  (.106) |
| Constant | -3.088***  (.440) | -2.958***  (.411) | -3.254***  (.494) | -2.761***  (.672) | -0.612*  (.252) | -0.530  (.432) |

*Notes.* * *p*<.050, ** *p*<.010, *** *p*<.001. Inequality condition is a dummy variable with a value of 1 in the inequality condition and 0 otherwise. Target wealth is a is a dummy variable with a value of 1 in the high wealth condition and 0 otherwise. Positive / negative words is the percentage of words falling into default positive / negative affect dictionaries of the software LIWC (Pennebaker et al., 2007).

**LIWC wealth category word codes**

In order to check the reliability of our coding, we created a new LIWC dictionary that included one- and two-word phrases that captured many of the synonyms of rich, middle class and poor that participants used in their descriptions. This dictionary is provided with the Supplementary documents. Examples include “1^st^ class”, “affluent”, “class one”, “2^nd^ tier”, “average class”, “middle status”, “class three”, “low class”, and “third tier” (the entire dictionary is provided as a supplementary document). We converted the percentage of words that fell into each wealth category into a dichotomous variable, assigning a value of 1 for any non-zero percentage, and 0 otherwise. While we included the most obvious synonyms in this dictionary, we could not include all possible references as participants showed remarkable variability in the ways in which they referred to the wealth categories and sometimes did so using more than 2 adjacent words (e.g., “I would spend time with people who were mostly similar in terms of level”). Additionally, it was possible that some of the dictionary phrases would lead to false positives (e.g., “at the gym, I attended the first class of the day”). These factors mean that this LIWC based approach has some inherent unreliability that was not present in our own codings. Nonetheless, reliability analysis revealed that the agreement between the LIWC codes and our original ones were for the most part moderate in size: across studies and targets, Group 1 Kappas ranged from .55 and .65, Group 2 Kappas ranged from .26 and .54, and Group 3 Kappas ranged from .50 to .60.

We replicated the contingency table and logistic regression analyses presented in the manuscript using the LIWC codes as dependent variables. This provided a very close replication of our original findings. Specifically, when Study 3a participants described their own life, those in the high inequality condition were more likely to mention group 2, their own group (51% versus 33%, B=0.76, odds ratio=2.14), χ^2^(1) = 7.60, p=.006, group 3, the poor group (30% versus 19%, odds ratio=1.57), χ^2^(1) = 3.43, p=.064, and group 1, the rich group (51% versus 29%, B=0.93, odds ratio=2.53), χ^2^(1) = 11.01, p=.001. When describing another citizen’s life, participants in the inequality condition were more likely to mention group 3 (37% versus 17%, B=1.08, odds ratio=2.95), χ^2^(1) = 11.29, p=.001; they were not, however, more likely to mention group 2 (14% versus 9%, B=0.45, odds ratio=1.58), χ^2^(1) = 1.13, p=.288, or group 1 (38% versus 31%, B=0.34, odds ratio=1.40), χ^2^(1) = 1.14, p=.287. At the same time, when the citizen was said to be a member of group 1 (rather than group 3), participants were more likely to reference group 1 (57% versus 13%, B=2.18, odds ratio=8.83), χ^2^(1) = 41.52 p<.001; they were no more likely to mention group 2 (10% versus 13%, B=-0.32, odds ratio=0.73), χ^2^(1) = 0.56, p=.455, or group 3 (25% versus 29%, B=-0.20, odds ratio=0.82), χ^2^(1) = 0.41, p=.525.

**Study 3b**

**Additional Manipulation Check**

We also examined perceptions of the wealth of the different wealth groups using a 2 (inequality condition: high or low) X 3 (wealth group: 1, 2 or 3) mixed ANOVA, with repeated measures on wealth group. The main effects of condition, F(1,412)=10.61, p=.001, η2=.03, and group, F(2,824)=772.26, p<.001, η2=.61, were again qualified by the two-way interaction, F(2,824)=46.21, p<.001, η2=.04. Simple effects analysis revealed that relative to participants in the low inequality condition participants in the high inequality condition perceived group 1 to be significantly more wealthy (low M=7.13, SD=1.52; high M=8.04, SD=1.98), F(1,412)=26.94, p<.001, η2=.06, and group 3 to be significantly less wealthy (low M=3.54, SD=1.66; high M=2.04, SD=2.31), F(1,412)=57.16, p<.001, η2=.12. In contrast, participants did not differ in perceptions of the wealth of their own group, group 2 (low M=5.30, SD=0.70; high M=5.28, SD=0.85), F(1,412)=0.06, p=.805, η2=.00.

**Attribute Importance Ratings**

Study 3b again measured participants’ ratings of the importance of a stranger’s morality (α=.77), warmth (α=.84), and competence (α=.86) in addition to the moral foundations. We again conducted a series of independent samples t-tests to compare participants’ ratings of the importance of knowing information that was more (versus less) strongly related to wealth as a function of experimental condition. We found that experimental condition had no significant effect on concern with a stranger’s morality, warmth or competence, all *t*(404)$\leq$1.17, *p*$\geq.$243), or their alignment with three of the five moral foundations, all *t*(404)$\leq$1.46, all *p*$\geq$.144 — the two exceptions are described below.

As in Study 3, there was a difference in ratings of the importance of being a loyal Bimboolean, as participants in the low inequality condition said that this information was more important than those in the high inequality condition (low *M*=3.60, *SD*= 1.07; high *M*=3.27, *SD*= 1.16), *t*(404)=3.05, *p*=.002. Participants in the low inequality condition also said that information about a stranger’s tendencies to uphold tradition was more important than those in the high inequality condition (low *M*=3.59, *SD*= 1.08; high *M*=3.25, *SD*= 1.17), *t*(404)=3.07, *p*=.002.

**Inequality as a Social Problem**

Table S6. Logistic regression coefficients of experimental condition and negative and positive affective terms on prevalence of wealth category words, *Own* Life.

|  | Group 1 | | Group 2 | | Group 3 | |
| --- | --- | --- | --- | --- | --- | --- |
|  | Step 1 | Step 2 | Step 1 | Step 2 | Step 1 | Step 2 |
| Inequality Condition | 0.338  (.205) | 0.208  (.213) | 0.347**  (.210) | 0.513*  (.217) | 0.829***  (.205) | 0.658**  (.214) |
| Positive words |  | -0.052  (.041) |  | -0.059  (.040) |  | -0.086*  (.042) |
| Negative words |  | 0 .265**  (.097) |  | 0.286*  (.115) |  | 0.436***  (.107) |
| Constant | -0.701***  (.149) | -0.624*  (.276) | 0.340*  (.143) | 0.454  (.277) | -0.791***  (.152) | -0.682*  (.281) |

*Notes.* * *p*<.050, ** *p*<.010, *** *p*<.001. Inequality condition is a dummy variable with a value of 1 in the inequality condition and 0 otherwise. Positive / negative words is the percentage of words falling into default positive / negative affect dictionaries of the software LIWC (Pennebaker et al., 2007).

Table S7. Logistic regression coefficients of experimental condition and negative and positive affective terms on prevalence of wealth category words, Citizen Life.

|  | Group 1 | | Group 2 | | Group 3 | |
| --- | --- | --- | --- | --- | --- | --- |
|  | Step 1 | Step 2 | Step 1 | Step 2 | Step 1 | Step 2 |
| Inequality Condition | 0.798**  (.272) | 0.809**  (.273) | 0.405  (.266) | 0.415  (.267) | 0.889***  (.234) | 0.962***  (.246) |
| Target Wealthy | 3.414***  (.307) | 3.502***  (.324) | 0.484  (.267) | 0.562*  (.278) | -2.160***  (.237) | -2.057***  (.248) |
| Positive words |  | 0.007  (.053) |  | 0.028  (.054) |  | 0.006  (.049) |
| Negative words |  | 0.136  (.122) |  | 0.170  (.111) |  | 0.567***  (.111) |
| Constant | -2.878***  (.316) | -3.130***  (.484) | -2.061***  (.259) | -2.463***  (.442) | 0.204  (.185) | -0.583  (.364) |

*Notes.* * *p*<.050, ** *p*<.010, *** *p*<.001. Inequality condition is a dummy variable with a value of 1 in the inequality condition and 0 otherwise. Target wealth is a is a dummy variable with a value of 1 in the high wealth condition and 0 otherwise. Positive / negative words is the percentage of words falling into default positive / negative affect dictionaries of the software LIWC (Pennebaker et al., 2007).

**LIWC wealth category word codes**

In order to check the reliability of our coding, we again used the wealth category LIWC dictionary to analyse the presence / absence of references to each wealth category in participants’ descriptions. When Study 3b participants described their own life, those in the high inequality condition were more likely to mention group 2, their own group (44% versus 27%, B=.76, odds ratio=2.13), χ^2^(1) = 13.81, p<.001, and group 3, the poor group (54% versus 34%, B=.83, odds ratio=2.29), χ^2^(1) =16.65, p<.001; they were not more likely to mention group 1, the rich group (40% versus 35%, B=.19, odds ratio=1.21), χ^2^(1) = 0.88, *p*=.347. When describing another citizen’s life, participants in the inequality condition were more likely to mention group 1 (43% versus 35%, B=.47, odds ratio=1.61), χ^2^(1) = 4.35, p=.037, and group 3 (38% versus 23%, B=.72, odds ratio=2.06), χ^2^(1) = 10.42, p=.001; they were not more likely to mention group 2 (13% versus 11%, B=.18, odds ratio=1.19), χ^2^(1) = 0.33, p=.567. At the same time, when the citizen was said to be a member of group 1 (rather than group 3), participants were more likely to reference group 1 (59% versus 17%, B=1.98, odds ratio=7.24), χ^2^(1) = 70.59, p<.001, and less likely to reference group 3 (22% versus 39%, B=-.80, odds ratio=0.45), χ^2^(1) = 12.92, p<.001; they were no more likely to reference group 2 (11% versus 12%, B=-.09, odds ratio=0.92), χ^2^(1) = 0.08, p=.780.

**A Note on Study 3a and 3b Exclusion Rates**

The exclusion rates for Studies 3a and 3b were indeed rather high, including 39% of those who embarked on Study 3a, and 38% of those who embarked on Study 3b. Participants were first excluded if they failed to complete all the measures (Study 3a = 82% of excluded, Study 3b = 37% of excluded), then excluded if they failed one or more of the attention checks (Study 3a = 11% of excluded, Study 3b = 37% of excluded), and finally excluded if their response to either of the free response questions was low quality (Study 3a = 7% of excluded, Study 3b = 25% of excluded). A low quality response was any response that did not follow the instructions. There were three main kinds of low quality responses: (1) those reproducing the question instructions, (2) those reproducing text from another source and (3) those consisting of fanciful / uninterpretable text. The first two categories were most common. It is notable that while the rate of exclusions was about the same in both studies, the basis for exclusions differed. This is most likely due to the fact that participants were paid more in Study 3b (US$2.50 versus US$1.00), which may have convinced otherwise low quality participants to complete all measures (especially the effortful open questions), albeit to a poor standard, meaning that they were subsequently excluded for other reasons.

The high exclusion rate could be problematic if there was evidence that exclusions were more likely in one condition than the other. There was no evidence of this. In Study 3a, we excluded 41.1% of participants in the equal condition and 36.1% in the unequal condition, χ^2^(1)=0.98, *p=*.323. In Study 3b, we excluded 39.5% in the equal condition and 36.0% in the unequal condition, χ^2^(1)=0.90, *p*=.342. Importantly, when we retained all participants who provided data on the importance of wealth attributes in the analysis (excluded participants were much more likely to provide this data than to provide a high quality response to the open question), we closely replicated the findings reported in the paper. Specifically, in Study 3a (*N*=290), we found that participants in the inequality condition placed more importance on knowing another’s salary, *t*(288)=2.39, *p*=.018, income group, *t*(288)=2.97, *p*=.003, and (marginally) occupation, *t*(288)1.67, *p*=.095; the difference in the importance in knowing about education that we originally observed was no longer significant, *t*(288)=1.21, *p*=.227. In Study 3b (*N*=601), we found that participants in the inequality condition placed more importance on knowing another’s salary, *t*(601)=2.06, *p*=.040 and income group, *t*(601)=3.99, *p*<.001.

The question then is why our exclusion rate is so high and (relatedly) whether it is higher than that experienced by other authors. The fact that the majority of participants were excluded because they either (1) failed to complete the open response questions or (2) provided a low quality response to these questions suggests that these questions played an important role. This is unsurprising when one considers that they require substantially more effort than responding to closed questions. Consistent with this possibility, in their blog describing replications of consumer psychology studies with MTurk participants (<http://datacolada.org/archives/category/data-replicada>), Nelson and Simmons report dropout rates of 22% to 42% for studies that include an open response writing task; when they considered the quality of the responses they excluded another 17% of the sample (<http://datacolada.org/89>). This at least suggests that our experience may not be that unusual among researchers using MTurk participants for studies that include free text responses. More importantly, there is no evidence that the rate of exclusions that we encountered invalidated the assumption of random allocation to condition.

**Study 5: Pre-Registered Survey Not Reported in Main Text**

**Method**

**Participants.** Our target sample size was 500 or more participants. This target was based on our Study 3a observation that the association between participants’ subjective perceptions that Bimboola was unequal and their endorsement of the importance of wealth-related attributes ranged from *r*=.131 to *r*=.205. Analysis with G*Power (Faul, Erdfelder, Buchner, & Lang, 2009) indicated that a sample of 500 participants would detect an effect of *r*=.131 or larger 80 percent of the time. We hired an Australian panel survey company to recruit a minimum of 500 members of the Australian community to complete the survey (reimbursement levels were arranged by the company). Of the 508 people who completed the survey, 10 were excluded for failing to provide meaningful responses to the open-ended questions. Therefore, the final sample for analysis purposes consisted of 498 Australians. Participants ranged in age from 18 to 60 or more years (modal age range was 40 to 49 years, *N*=94), and just over half were female (*N*=260; male *N*=234, other responses *N*=4). Over half of participants were in part- or full-time employment (full-time *N*=146; part-time *N*=129; not employed *N*=110; retired *N*=113) and the majority had post-secondary education (*N*=350). There was a wide spread in self-reported socio-economic status, with 32 percent below average (scores 1–4), 44 percent about average (scores 5 or 6), and a quarter above average (scores 7–10) on the 10-point SES ladder.

**Measures.** Participants were first asked to respond to 11 items that assessed their beliefs about the way in which wealth is distributed in Australia, including 3 items that measured *subjective inequality* (α=.78): “Overall, how small or large is the wealth gap between the poorest and the wealthiest people in Australia?”, “In your view, to what extent has the wealth gap between the poorest and the wealthiest people in Australia decreased or increased over the last decades?” and “How much do you agree that the gap between rich and poor Australians is so large that it is as if they live in different worlds?” Participants responded to these items on 7-point scales, where 1=low perceived inequality and 7=high perceived inequality. As an alternative measure of perceived inequality, we also asked participants to respond to Sprong et al.’s (2019) measure of *subjective Gini*. This involved asking participants to think of 100 Australian citizens and to split them between the following wealth categories: “very poor”, “poor”, “average in wealth”, “wealthy”, “very wealthy.”

After this, participants were asked to respond to demographic questions about their employment status, personal and household income, education and perceived socio-economic status. They were then asked to respond to the dependent variables. These included the open response and attribute ratings tasks that were described in Studies 3 and 4. These were adapted to make sense in the current context (i.e., asking about their life in Australia, or to imagine meeting another Australian who is poor or wealthy — wealth group randomly allocated), but were otherwise identical.

**Results**

**Coding.** Using the approach described in the previous studies, the first author and a research assistant again independently coded participants’ spontaneous use of wealth categories in their free responses describing their own life and that of another citizen. The first coding round resulted in reasonable levels of agreement for rich and poor categories (own life Kappas: 0.52 – 0.62; citizen life Kappas: 0.55 – 0.65) but not for the middle class (own life Kappa: 0.26; citizen life Kappa: 0.13). The second coding round produced reasonable to high levels of agreement (own life Kappas: 0.82 – 0.94; citizen life Kappas: 0.65 – 0.95). Any remaining disagreements were resolved by the first author.

**Wealth references.** To test H1, we used logistic regressions to examine participants’ tendencies to spontaneously reference wealth categories when describing their own life as a function of their subjectively perceived inequality for each wealth category in turn. This revealed that participants who believed that Australia was a more unequal society were significantly more likely to mention the poor (B=0.33, odds ratio=1.40), χ^2^(1) = 8.97, *p*=.003, and marginally more likely to mention the rich (B=0.36, odds ratio=1.43), χ^2^(1) = 2.85, *p*=.091. They were no more likely to mention the middle class (B=0.05, odds ratio=1.05), χ^2^(1) = 0.12, *p*=.728. Repeating this regression with subjective Gini in place of subjective inequality produced a similar pattern of findings (see Supplementary materials).

To examine how participants’ subjective perception of inequality affected the prevalence of wealth category references in descriptions of another citizen’s life, we again ran logistic regressions with subjective inequality and target wealth (high wealth=1, otherwise 0) as predictors for each wealth category in turn. These analyses revealed that participants who perceived that inequality was higher were descriptively more likely to reference wealth categories (i.e., odds ratios exceeded 1), but these differences were not significant: rich B=0.08, odds ratio=1.09, χ^2^(1) = 0.50, *p*=.481, poor B=0.06, odds ratio=1.06, χ^2^(1) = 0.30, *p*=.584, and middle class B=0.10, odds ratio=1.10, χ^2^(1) = 0.21, *p*=.646. At the same time, when the citizen was said to be rich (rather than poor), participants were more likely to reference the rich (B=3.19, odds ratio=24.35), χ^2^(1) = 84.48, *p*<.001, and less likely to reference the poor (B=-2.71, odds ratio=0.07, χ^2^(1) = 75.57, p<.001; references to the middle class were unaffected (B=0.49, odds ratio=1.64, χ^2^(1) = 1.27, p=.261. Repeating these regressions with subjective Gini as a predictor produces an identical pattern of findings (see Supplementary materials).

**Wealth attribute importance.** In order to test H2, we conducted a series of bivariate correlations to examine participants’ ratings of the importance of information that was more (versus less) strongly related to wealth as a function of perceived inequality. Unexpectedly, we found that the more participants perceived a wealth gap, the less important they said it was to know a stranger’s salary (inequality *r*=-.10, *p*=.032; Gini *r*=-.01, *p*=.896) and education (inequality *r*=-.13, *p*=.005; Gini *r*=-.05, *p*=.266). These participants also said it was less important to know a person’s religion (inequality *r*=-.19, *p*=.001; Gini *r*=-.05, *p*=.238). There were no significant associations with the perceived importance of the other demographic variables, all *p*$\geq$.106.

**Wealth category references**

We repeated the logistic regressions described in the paper with subjective Gini in place of subjective inequality. This produced a similar pattern of findings. Specifically, participants who believed that Australia was a more unequal society were significantly more likely to mention the poor (B=4.57, odds ratio=96.55), χ^2^(1) = 5.65, *p*=.017. However, they were not significantly more likely to mention the rich (B=1.88, odds ratio=6.52), χ^2^(1) = 0.30, *p*=.585 or the middle class (B=-1.06, odds ratio=0.35), χ^2^(1) = 0.17, *p*=.678.

Substituting subjective Gini perceptions into the analyses of wealth category references when describing another citizen’s life produced the same pattern of findings as those discussed in the paper. In particular, there were no significant differences in tendency to reference wealth categories as a function of participants’ subjective Ginis: rich B=-0.834, odds ratio=0.43, χ^2^(1) = 0.17, *p*=.685, poor B=1.21, odds ratio=3.36, χ^2^(1) = 0.35, *p*=.554, and middle class B=-1.09, odds ratio=0.34, χ^2^(1) = 0.09, *p*=.769.

**Attribute importance ratings.**

We conducted a series of bivariate correlations to examine participants’ ratings of the importance of information that was more (versus less) strongly related to wealth as a function of perceived inequality. There were no significant associations with a stranger’s morality or competence, all *p*$\geq$.200, or their alignment with three of the five moral foundations, significant, all *p*$\geq$.106). However, participants who perceived that Australia was more unequal placed greater importance in knowing the citizen’s warmth (inequality *r*=.10, *p*=.033; Gini *r*=.12, *p*=.007) and in their alignment with the moral foundations of care / harm (inequality *r*=.19, *p*=.001; Gini *r*=.13, *p*=.004) and purity (inequality *r*=-.15, *p*=.001; Gini *r*=.05, *p*=.249). Interestingly, these individuals were more likely to say that it was more important to know about another person’s warmth and their alignment with the care / harm morality foundation. Although the unexpected nature of these associations suggest that caution should be exercised in interpreting them, this may reflect a greater concern with other’s social intentions in more unequal social contexts.

**Discussion**

This survey provided partial support for H1. In particular, it revealed that participants who perceived that there was greater wealth inequality in Australia were more likely to mention the poor and (marginally) the rich when discussing their lives. Although this pattern was descriptively consistent when we looked at participants’ references to wealth categories when discussing another person, the effects were not significant. This raises the possibility that the size of the effects is somewhat smaller than we anticipated, and therefore that the current study was under-powered. In contrast, this survey did not provide any support for H2, as if anything greater perceived inequality was associated with a tendency to say that it was less important to have information on several demographic attributes, including some related to income. Given these somewhat inconsistent findings, it is worth noting that Study 2 did not find any evidence that inequality was associated with wealth category references in Australia’s media; greater evidence in line with our hypotheses may have been observed in countries that did provide evidence for such effects (e.g., the UK).

**References**

Brambilla, M., Sacchi, S., Rusconi, P., Cherubini, P., & Yzerbyt, V. Y. (2012). You want to give a good impression? Be honest! Moral traits dominate group impression formation. *British Journal of Social Psychology*, *51*(1), 149-166.

Yilmaz, O., Harma, M., Bahçekapili, H. G., & Cesur, S. (2016). Validation of the moral foundations questionnaire in Turkey and its relation to cultural schemas of individualism and collectivism. *Personality and Individual Differences*, *99*, 149-154.

Haidt, J., Graham, J., & Joseph, C. (2009). Above and below left–right: Ideological narratives and moral foundations. *Psychological Inquiry*, 20(2-3), 110-119.
